# Sirt6 enhances macrophage lipophagy and improves lipid metabolism disorder by regulating the Wnt1/β-catenin pathway in atherosclerosis

**DOI:** 10.1186/s12944-023-01891-3

**Published:** 2023-09-22

**Authors:** Tingting Wang, Zheng Cheng, Ran Zhao, Jin Cheng, He Ren, Pengke Zhang, Pengyun Liu, Qimeng Hao, Qian Zhang, Xiaolei Yu, Dongdong Sun, Dongwei Zhang

**Affiliations:** 1 Department of Cardiology, Tangdu Hospital, Fourth Military Medical University, Xi’an, 710032 China; 2 Department of Cardiology, Xijing Hospital, Fourth Military Medical University, Xi’an, 710032 China

**Keywords:** Atherosclerosis, Lipid metabolism, Macrophage lipophagy, Foam cells, Sirt6

## Abstract

Lipid metabolism disorders are considerably involved in the pathology of atherosclerosis; nevertheless, the fundamental mechanism is still largely unclear. This research sought to examine the function of lipophagy in lipid metabolism disorder-induced atherosclerosis and its fundamental mechanisms. Previously, Sirt6 has been reported to stimulate plaque stability by promoting macrophage autophagy. However, its role in macrophage lipophagy and its relationship with Wnt1 remains to be established. In this study, ApoE^−/−^: Sirt6^−/−^ and ApoE^−/−^: Sirt6Tg mice were used and lipid droplets were analysed via transmission electron microscopy and Bodipy 493/503 staining in vitro. Atherosclerotic plaques in ApoE^−/−^: Sirt6^−/−^ mice showed greater necrotic cores and lower stability score. Reconstitution of Sirt6 in atherosclerotic mice improved lipid metabolism disorder and prevented the progression of atherosclerosis. Furthermore, macrophages with Ac-LDL intervention showed more lipid droplets and increased expression of adipophilin and PLIN2. Reconstitution of Sirt6 recruited using SNF2H suppressed Wnt1 expression and improved lipid metabolism disorder by promoting lipophagy. In addition, downregulation of Sirt6 expression in Ac-LDL-treated macrophages inhibited lipid droplet degradation and stimulated foam cell formation. Innovative discoveries in the research revealed that atherosclerosis is caused by lipid metabolism disorders due to downregulated Sirt6 expression. Thus, modulating Sirt6’s function in lipid metabolism might be a useful therapeutic approach for treating atherosclerosis.

**Supplementary Information:**

The online version contains supplementary material available at 10.1186/s12944-023-01891-3.

## Introduction

The major complication of atherosclerotic cardiovascular disease (ASCVD) is acute myocardial infarction, which is one primary contributor to disability and death worldwide. Given the limited availability of therapeutic strategies that can stabilise vulnerable atherosclerotic plaques, it is necessary to develop novel strategies for preventing the formation of plaques instead of treating the established plaques and acute cardiovascular events [1, 2]. Atherosclerosis primarily results from redundant lipid accumulation in macrophages after dysregulated metabolism and macrophage lipid transport, resulting in the generation of foam cells [3]. Exacerbated and unstable plaques may form when foam cells are remarkably retained in the subintima, allowing for the formation and development of plaques with a high accumulation of necrotic pools [4]. However, to date, specific strategies for preventing lipid metabolism disorders, which can inhibit foam cell formation in patients with atherosclerosis, have received little attention.

Macrophages uptake lipoproteins and the clearance of lipids from macrophage cells determine the formation of foam cells [5, 6]. Low-density lipoprotein cholesterol (LDL-C) and postprandial triglycerides (TGs) are found in atherosclerotic lesions and macrophages isolated from these lesions [7, 8]. In addition, they are the main risk factors for ASCVD in humans [9, 10]. Previous studies have suggested that among the several mechanisms underlying macrophage foam cell formation is the oxidized LDL (ox-LDL)-scavenger receptor pathway. Recent studies have also reported the TG-rich lipoprotein (TGRL)-LPL-very-low-density lipoprotein (VLDL) pathway as another mechanism [11]. As a member of the LDL receptor family, the VLDL receptor is capable of recognizing apolipoprotein (apo)E and binding to TGRL, both of which play a vital role in macrophage foam cells formation [12, 13]. The reverse cholesterol transport (RCT) and lipid interchange between VLDL and LDL are mediated by high-density lipoprotein (HDL). HDL is also considered to be implicated in the development of atherosclerotic plaques [14]. Abundant TGs and LDL cholesterol disturb the structure of HDL, thus contributing to lipid metabolism disorder in macrophages [15]. Foam cells are macrophages that have cytoplasmic lipid droplets (LDs) and are bound by membranes [11]. LDs are specialised organelles in which excessive TGs and esterified cholesterol are stored. For this reason, they might be a potential therapeutic target in the fight against atherosclerosis [11, 16]. Although extensive research has been done to identify the pathways contributing to foam cell formation and plaque instability, the molecular mechanisms underlying LDs metabolism that regulate the formation of foam cells remain largely unknown. Autophagy is a well-studied mechanism that all eukaryotic cells use for the turnover of their proteins and organelles [17]. In recent years, researchers have discovered that autophagy also participates in the catabolism of lipids, also known as “lipophagy” [18]. In lipophagy, LDs are selectively delivered to lysosomes for degradation [18, 19] to produce fatty acids (FAs) and free cholesterol [20]. Researchers have demonstrated the crucial function of lipophagy in regulating cholesterol efflux from macrophages via lysosome degradation [19]. To our knowledge, nonetheless, no research has revealed if targeting lipophagy is a viable technique to enhance the degradation of LDs and improve dysregulated lipid metabolism in macrophages, thus reducing the production of foam cells and preventing the onset of atherosclerosis.

Sirt6, one of seven sirtuins (Sirt1-Sirt7), is a pleiotropic regulator of genome stability, glucose metabolism, tumour suppression and the organismal lifespan [21]. In the previous investigation, Sirt6 has been reported to promote macrophage autophagy, thus promoting the stability score of established atherosclerotic plaques [22]. Nevertheless, there is little research reported that whether Sirt6 may prevent the formation of foam cells and plaques by regulating dysregulated lipid metabolism via promoting the degradation of LDs in macrophages through lipophagy. In addition, the potential underlying mechanisms remain to be defined. To evaluate Sirt6 as a possible therapeutic target for promoting lipid droplets degradation thus preventing foam cell formation, the significance of the Sirt6/Wnt1/β-catenin pathway was emphasized in the investigation. The innovative research revealed that modulating Sirt6’s function in lipid metabolism might be a useful therapeutic approach for treating atherosclerosis.

## Materials and methods

### Animal handling

Macrophage-specific Sirt6-deficient mice were produced using the standard Cre-LoxP-based targeting approaches. Macrophage cell-specific Sirt6-conditional-knockout (Sirt6^−/−^: Lyz2^cre/+^) mice (Littermate: C57BL/6J Background; Jackson Laboratories, USA) were produced by mating Sirt6^flox/flox^ mice with Lyz2^cre^ knock-in mice. For producing ApoE^−/−^: Sirt6^−/−^ mice, Sirt6^flox/flox^ mice were initially crossed with ApoE^−/−^ mice to generate ApoE^−/−^: Sirt6^flox/flox^ mice, and subsequently, ApoE^−/−^: Sirt6^flox/flox^ mice were crossed with Lyz2^cre^ mice to produce ApoE^−/−^: Sirt6^−/−^ mice. As described previously [22], ApoE^−/−^ mice were injected with the AAV-Sirt6 virus to produce ApoE^−/−^: Sirt6Tg mice. ApoE^−/−^ mice were crossed with Atg5^flox/flox^ mice to produce ApoE^−/−^: Atg5^flox/flox^ mice, which were crossed with Lyz-2^cre^ mice to produce ApoE^−/−^: Lyz2^cre^: Atg5^−/−^ mice [23]. For 16 weeks, these mice were given a western diet consisting of 0.2% bile acid salt, 1.25% cholesterol, and 15% fat. Intraperitoneal administration of 0.5 mg/kg rapamycin and 10 mg/kg/day of the autophagy inhibitor 3-MA was done to a group of ApoE^−/−^ mice fed a Western diet [24]. All these mice were housed in conformity with the protocols established by the Institutional Animal Care and Use Facility of the Fourth Military Medical University (Approval ID: 2014189).

### Haematoxylin–eosin and Masson staining

Following a period of 16 weeks, the mice were euthanized, and their aortas were isolated. Staining with haematoxylin–eosin (H&E) allowed for the visualisation of atherosclerotic lesions, and Masson staining was used to measure the content of collagen following the guidelines provided by the manufacturer (Wuhan Servicebio Technology Co., Ltd.). For better visualisation of plaque size and collagen composition, H&E and Masson trichrome staining were performed on serial plaque sections, correspondingly. Plaque areas and collagen components were quantified utilising the ImageJ software from the National Institutes of Health by measuring the ratio of plaque area and Masson trichrome staining area to the total cross-sectional vascular wall area.

### Oil red O (ORO) staining

The necrotic core in plaques was assessed via ORO staining. After fixing enough aortic sections with PFA at a concentration of 4%, washing them with isopropanol at a concentration of 60%, and then staining them with ORO for 15 min, haematoxylin was used as a counterstain. After treating macrophages with Ac-LDL for 30 h, they were subsequently treated with rapamycin (ab120224, Abcam) to promote autophagy or exposed to 3-MA (Sigma-Aldrich Shanghai Trading Co., Ltd.) to inhibit autophagy. Thereafter, they were subjected to 20 min staining with ORO at 37 °C after being fixed with 4% PFA and rinsed in PBS By employing an optical microscope with an imaging system, the necrotic core content was calculated with the aid of the Image-Pro Plus program.

### Immunofluorescence (IF) and immunohistochemical (IHC) staining

After a 30-min blocking with BSA, slices of the aorta were treated at 4 °C for an entire night with mouse monoclonal anti-Sirt6 (ab191385, 1:100; Abcam), α-smooth muscle actin (SMA) (GB13044, 1:200; Servicebio), and anti-CD68 (GB11067, 1:100; Servicebio). Thereafter, rabbit anti-mouse secondary antibody (1:1,000; Abcam) was used to stain the sections for 1 h at room temperature (RT) before rinsing and mounting them with a medium containing 4,6-diamidino-2-phenylindole (DAPI). Haematoxylin was used to stain the nucleus. Subsequently, an upright fluorescent microscope and an Olympus FV1000 laser confocal microscope were used for the purpose of visualising the sections.

### Cell culture

The American Type Culture Collection (ATCC) supplied the murine macrophage cell line RAW264.7. The cells were grown in a humidified environment (37 °C, 5% CO_2_) after being plated on a 6-well plate (1.5 × 10^6^ cells/well) in RPMI 1640 medium (Hyclone, UT, USA) with 10% FBS (Hyclone, UT, USA). Subsequently, the cells were incubated with 50-ug/mL Ac-LDL (Cat No: 20604ES05; Qcbio Science Technologies Co., Ltd.) for 30 h to induce foam cell formation [16]. Finally, the cells were treated with 250-nM salinomycin (Sigma) for 24 h to inhibit Wnt1 signalling [25].

### Adenovirus management

As described previously [22], adenoviruses expressing a short hairpin (sh) RNA directed against Sirt6 (Ad-sh-Sirt6), adenoviruses expressing a short hairpin (sh) RNA directed against SNF2H (Ad-sh-SNF2H), those harbouring Sirt6 (Ad-Sirt6), adeno-associated virus (AAVSirt6), and control vectors (Ad-LacZ and Ad-sh-LacZ) were procured from Hanbio Technology Ltd (Shanghai, China). Adenoviruses were used to transduce the cells following the protocol provided by the manufacturer. Treatment with or without Ac-LDL (50 μg/mL) was done to the macrophages for 30 h, immediately after 12 h. During the last 1 h of Ac-LDL treatment, the cells were either treated or untreated with bafilomycin A1 (20 nmol/L). The multiplicity of infection (MOI)for adenoviruses was 80:1, and the titre was 1.26 × 10^10^ PFU/mL.

### Transmission electron microscopy

Collecting the Ac-LDL-loaded macrophages, transferring them to 1.5 mL Eppendorf tubes, and centrifuging them at a rate of 1806 g for 15 min were done in the same manner as was reported before [26]. Typical LDs were examined with a JEM-2000EX transmission electron microscope operating at 60 kV after postfixing and staining the macrophages with lead citrate and uranium acetate.

### Fluorescence microscopy

Bodipy 493/503 was used to stain lipid droplets in macrophages. Live-cell imaging was performed by staining Ac-LDL-loaded macrophages with Bodipy (10 ug/ml) for 30 min in the presence or the absence of LysoTracker Red (50 nM). Cells were stained with Bodipy, LysoTracker and LC3 to determine the location of lysosomes, LDs, and autophagosomes. In addition, Ac-LDL-loaded macrophages underwent co-localisation of the LAMP1-positive lysosomes with LDs coat protein adipophilin. The upright fluorescent microscope and Olympus FV1000 laser confocal microscope were used for observing all images.

### Co-IP

Refer to ESM methods and materials (Proteintech Immunoprecipitation Kit, Cat No.: KIP-2).

### Western blotting

Aortic tissue was processed for protein extraction, and macrophages were grown as directed by the manufacturer (Invitrogen, Carlsbad, CA, USA) [22] and quantified using the Bradford assay (Bio-Rad Laboratories, Hercules, CA, USA). Subsequently, proteins were isolated via SDS-PAGE using antibodies against Beclin1 (ab62472; Abcam), SNF2H (ab72499; Abcam), P62 (ab91526; Abcam), LC3A/B (ab128025; Abcam), Wnt1 (ab15251; Abcam), Sirt6 (ab191385; Abcam), β-catenin (ab32572; Abcam), GAPDH (ab181602; Abcam), LAMP1 (ab25245; Abcam), PLIN2 (ab108323; Abcam) and Adipophilin (ab108323; Abcam). A chemiluminescence system (Amersham Bioscience, Buckinghamshire, UK) was used to visualize the blots, and the Image-Pro Plus program (Media Cybernetics, MD Rockville, USA) was employed for quantifying the images.

### siRNA transfection

ATG5 (Origene, SR407903) and ATG7 (Origene, SR419264) siRNAs were transfected into macrophages in a 24-well plate. The recommended Lipofectamine® 2000 CD amount was used as a starting point. Unless otherwise specified, all amounts and volumes are administered on a per-well basis. Two tubes were set up a day before transfection commenced. RNAiMAX Transfection Reagent and Dulbecco’s modified Eagle’s medium (DMEM) were placed in one tube, whereas DMEM and siRNA were placed in the other. At RT, the two tubes were merged and left to incubate for five minutes. Forty-eight hours following transfection with ATg5 and ATg7 siRNAs, the macrophages were subjected to Ac-LDL treatment.

### Statistics

Continuous variables were presented as mean ± standard error of the mean (SEM). When comparing data across various groups, we used either the unpaired Student’s t-test or one-way ANOVA followed by Fisher’s post hoc test. Overall, this research used two-sided tests. A *P*-value of < 0.05 indicated a significance level. Image-Pro Plus was used for all analyses of statistical data (Media Cybernetics, Rockville, MD, USA).

## Results

### Sirt6 was observed in macrophage-derived foam cells

**Fig. 1:**
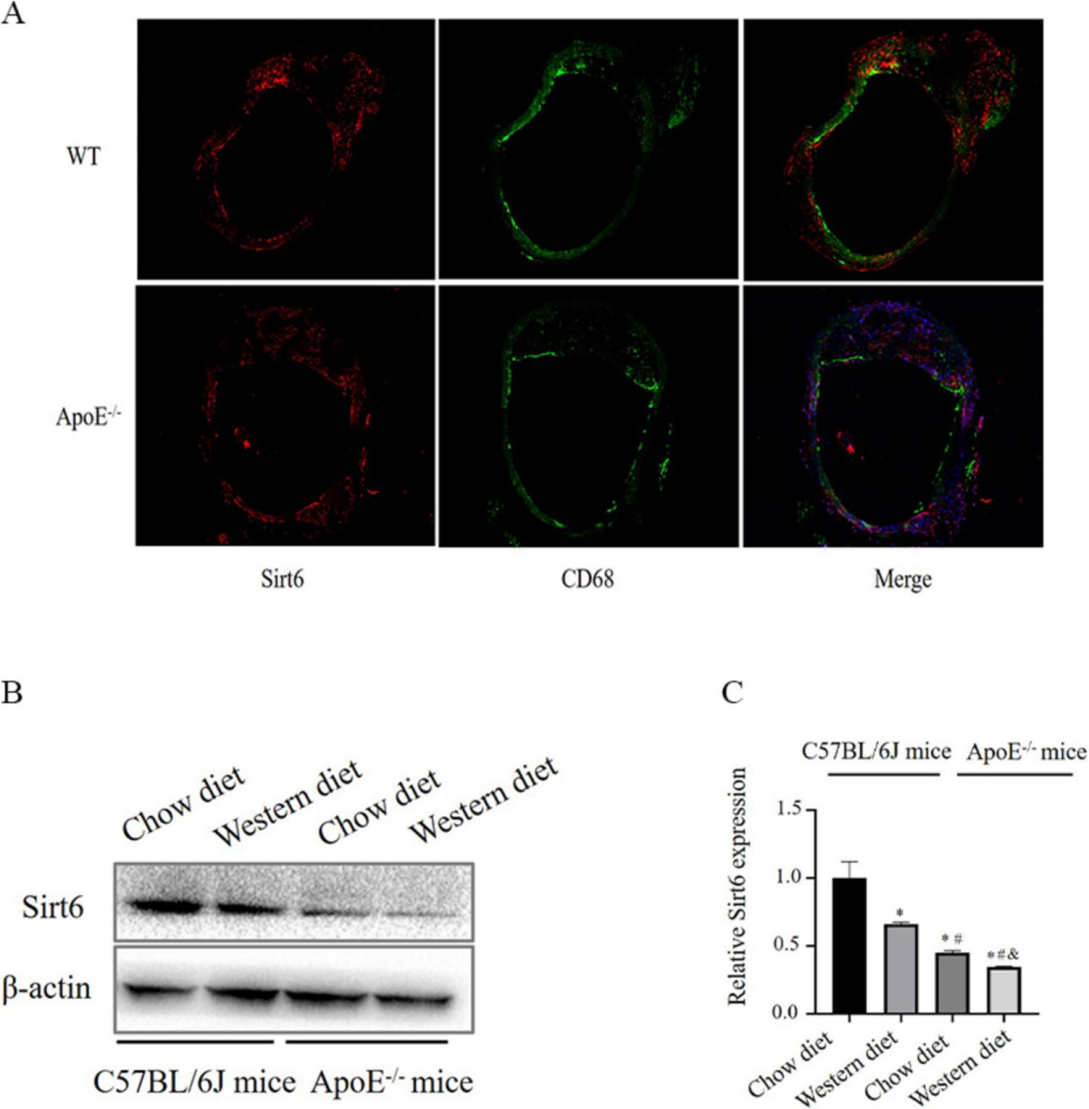
Sirt6 was observed in macrophage-derived foam cells. **A** WT mice and ApoE^−/−^ mice (*n* = 6) with atherosclerotic plaques were given a western diet (WD) for 16 weeks, stained (red) for Sirt6 and CD68 (green), and co-localised (yellow merging); DAPI staining was performed on the nuclei (blue). **B** Immunoblots demonstrating the expression of Sirt6 in the aorta obtained from wild-type C57BL/6J mice and ApoE^−/−^ mice given a chow diet and western diet (*n* = 10). **C** Quantification of Sirt6 expression in each group (*n* = 10) (One way ANOVA showed that there was statistical significance among groups. ^*^P < 0.05 versus (vs) WT mice [*n* = 10]; ^#^*P* < 0.05 vs ApoE^−/−^ mice [*n* = 10]; ^$^*P* < 0.05 vs ApoE^−/−^: Sirt6^−/−^ mice [*n* = 10]

The expression of Sirt6 in macrophage foam cells was examined in atherosclerotic plaques from ApoE^−/−^ mice fed a western diet for 16 weeks to determine if macrophagic Sirt6 involved in the progression of arteriosclerosis. Macrophages in plaques were stained with antibodies against Sirt6 and CD68. In ApoE^−/−^ mice with atherosclerotic plaques, Sirt6 was detected by immunofluorescence staining in CD68-labelled macrophage foam cells (Fig. 1A), while in the WT mice, neither Sirt6 nor CD68 was detected (Fig. 1A). Furthermore, a normal and a Western diet was given to ApoE^−/−^ and wild-type (WT) C57BL/6J mice. The Western diet has been reported to construct atherosclerosis in vivo. Immune blotting (Fig. 1B-C) revealed that in contrast with WT mice fed a normal diet and a western diet, and the ApoE^−/−^ mice fed a normal diet, the level of Sirt6 expression in ApoE^−/−^ mice receiving a western diet was significantly decreased. As shown by these findings, Sirt6’s expression may be related to lipid accumulation in macrophages, it is involved in the progression of atherosclerosis.

### Autophagy contributes to lipid metabolism in atherosclerosis

**Fig. 2:**
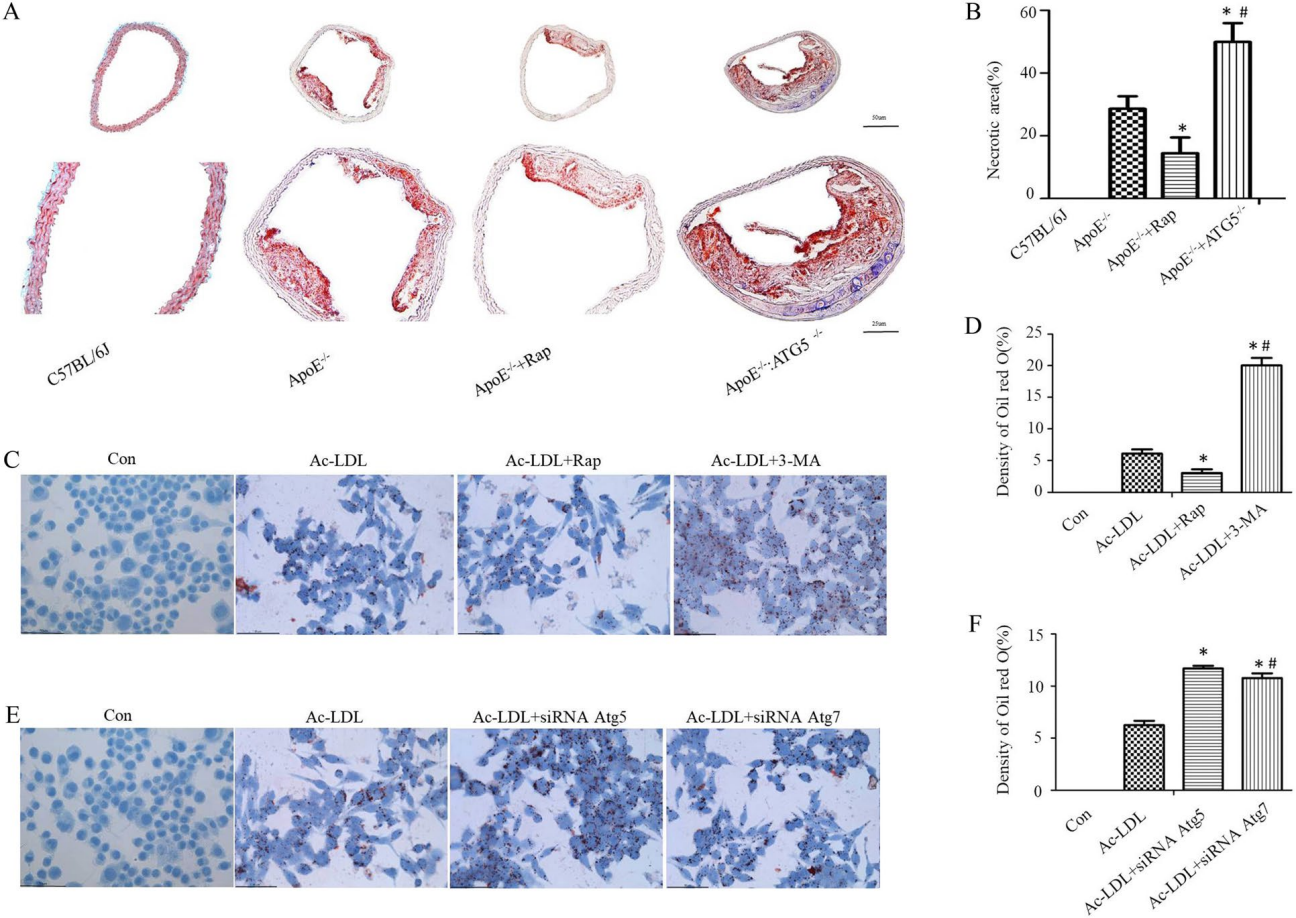
Autophagy attenuates the area of necrotic core in plaques and inhibits the formation of foam cells. ApoE^−/−^, ApoE^−/−^ + Rap and ApoE^−/−^: ATG5^−/−^ mice were fed for 16 weeks with western diet (*n* = 10) to trigger atherosclerosis. **A** Lipids in the aortic root were stained with oil red O. **B** Assessment of the necrotic area quantitatively. Data are expressed as mean ± SE (One way ANOVA showed that there was statistical significance among groups. ^*^P < 0.05 vs. ApoE^−/−^ mice [*n* = 10]; ^#^*P* < 0.05 vs. ApoE^−/−^ + Rap on a western diet [*n* = 10]; Student’s t-test showed that there was statistical significance between ApoE^−/−^ + Rap and ApoE^−/−^ mice, *P* < 0.01; in addition, there was statistical significance between ApoE^−/−^: ATG5^−/−^ mice and ApoE^−/−^ mice, *P* < 0.01). **C**–**E** Autophagy inhibited the conversion of macrophages into foam cells. The cells were activated by 50-μg/mL Ac-LDL in the presence of rapamycin, 3-MA, siRNA Atg5 and siRNA Atg7 for 30 h and subsequently stained with oil red O. **D**, **F** Quantitative analysis of the intensity of oil red O staining. Data are presented as mean ± SEM (One way ANOVA showed that there was statistical significance among groups. ^*^P < 0.05 vs. the Ac-LDL group; ^#^*P* < 0.05 vs. the Ac-LDL + Rap and Ac-LDL + siRNA Atg5 groups [*n* = 6 independent experiments])

WT, ApoE^−/−^, ApoE^−/−^ + Rap and ApoE^−/−^: ATG5^flox/flox^: Lyz2-cre mice were provided with a western diet for 16 weeks, and atheroma formation was analysed in them. Autophagy was hypothesised to exert a remarkable function in lipid metabolism. To validate this hypothesis, ORO staining was performed (en face lipid staining) and found that the total atherosclerotic lesion area was reduced by 49% in the aortic lesions of ApoE^−/−^ mice following treatment with rapamycin (14.49% ± 2.04% in ApoE^−/−^ + Rap versus 28.62% ± 1.63% in ApoE^−/−^ mice; *P* < 0.01, Student’s t-test; Fig. 2A–B). However, a 43% increase in lesion areas was observed in ApoE^−/−^: ATG5^−/−^ mice (49.91% ± 2.45% in ApoE^−/−^: ATG5^−/−^ versus 28.62% ± 1.63% in ApoE^−/−^ mice; *P* < 0.01, Student’s t-test; Fig. 2A–B).

Because modified LDL can lead to foam cell formation [27], acetylated LDL (Ac-LDL) was employed to increase the levels of intracellular lipids in macrophages. To determine whether autophagy regulates lipid levels in foam cells, it was pharmacologically inhibited using 3-methyladenine (3-MA) and stimulated using rapamycin. ORO staining showed that 3-MA significantly increased lipid content, whereas rapamycin remarkably decreased lipid content in foam cells (Fig. 2C–D). In addition, knockdown of the autophagy-related gene Atg5 siRNA (siAtg5, 100 nM) or Atg7 siRNA (siAtg7, 100 nM) in macrophages also increased lipid levels (Fig. 2E–F). The effects of Atg5 siRNA and Atg7 siRNA were proved by western blot (Supplementary Fig. 1). These findings indicate that autophagy may contribute to lipid droplets breakdown.

### Lipid droplet catabolism is impaired in Sirt6^−/−^ macrophage foam cells

**Fig. 3:**
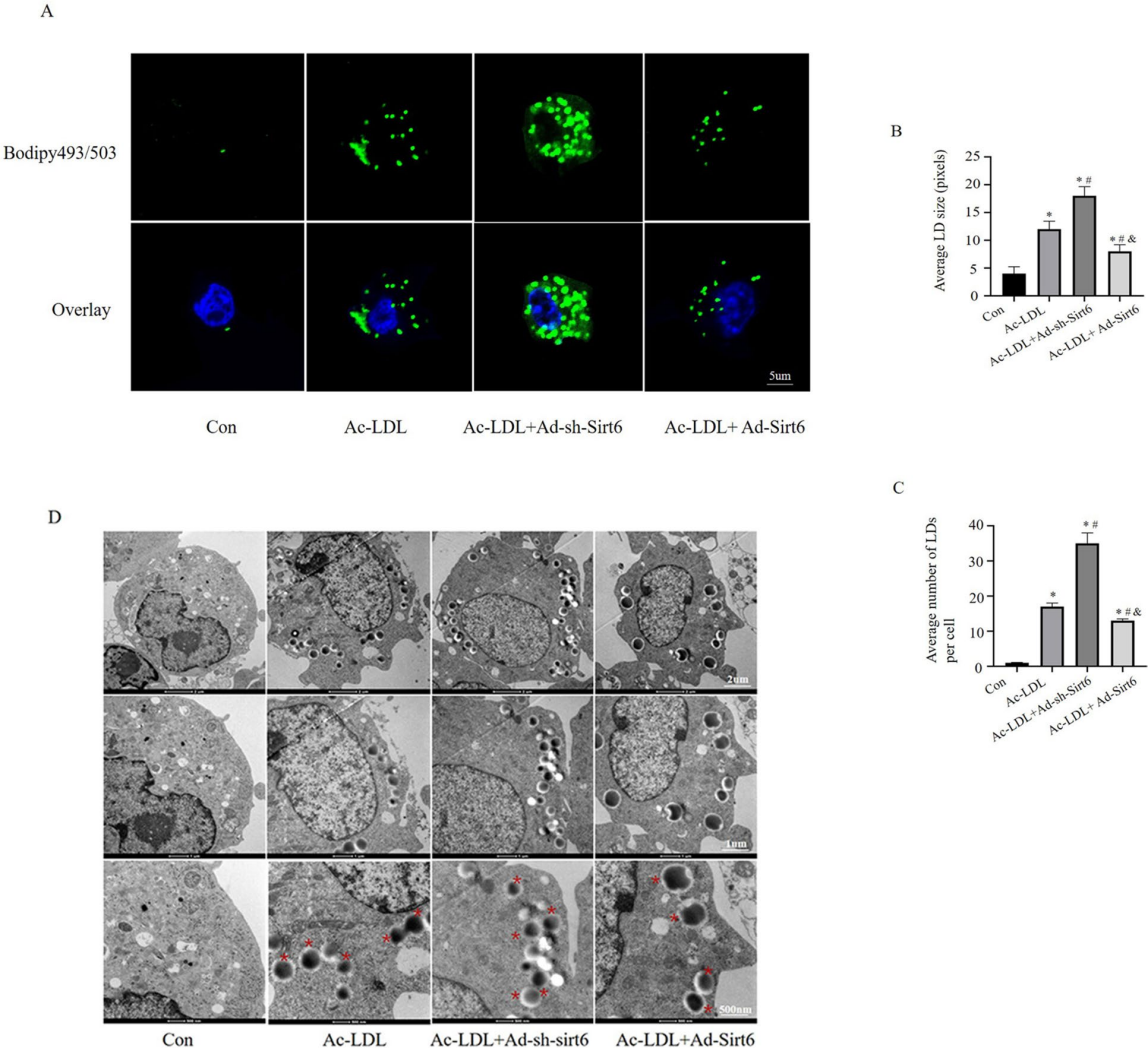
The inhibition of Sirt6 leads to an increase in lipid droplet accumulation in macrophages. **A** Number of lipid droplets observed in macrophages untreated (none) or treated with Ac-LDL and cultured in Ad-sh-Sirt6 or Ad-Sirt6 medium; BODIPY 493/503 was used to stain the LDs in macrophages. **B** The average number of LDs; **C** Average LD size(pixels), data are presented as mean ± SEM (One way ANOVA showed that there was statistical significance among groups, ^*^P < 0.05 vs the control group; ^#^*P* < 0.05 vs the Ac-LDL group; ^&^*P* < 0.05 vs the Ac-LDL + Ad-sh-Sirt6 group). **C** Visualisation of lipid droplets via electron microscopy (*n* = 6 independent experiments)

Furthermore, the effect of Sirt6 on lipid droplets accumulation in macrophage foam cells was evaluated. Since Sirt6 is essential for LDs generation and lipid storage in both adipose tissue and hepatocytes [28], whether Sirt6-defective macrophages might accumulate LDs in macrophages treated with Ac-LDL was evaluated. Confocal microscopy of cells in a heat chamber maintained at 37° C allowed for real-time visualisation of LDs after they had been stained with Bodipy 493/503 to distinguish them from neutral lipids. In macrophages, LDs appeared as an unidentified spherical organelle, and over time, a “ring” stained for neutral lipids formed surrounding the organelle. In addition, both increased number and size of LDs were observed in macrophages transfected with Ad-sh-Sirt6 (Fig. 3A–B), which was verified via electron microscopy (Fig. 3C). These findings suggest that absence of Sirt6 remarkably enhances the accumulation of intracellular lipid droplets in macrophages.

### Sirt6-mediated lipophagic flux modulates foam cell lipolysis

**Fig. 4:**
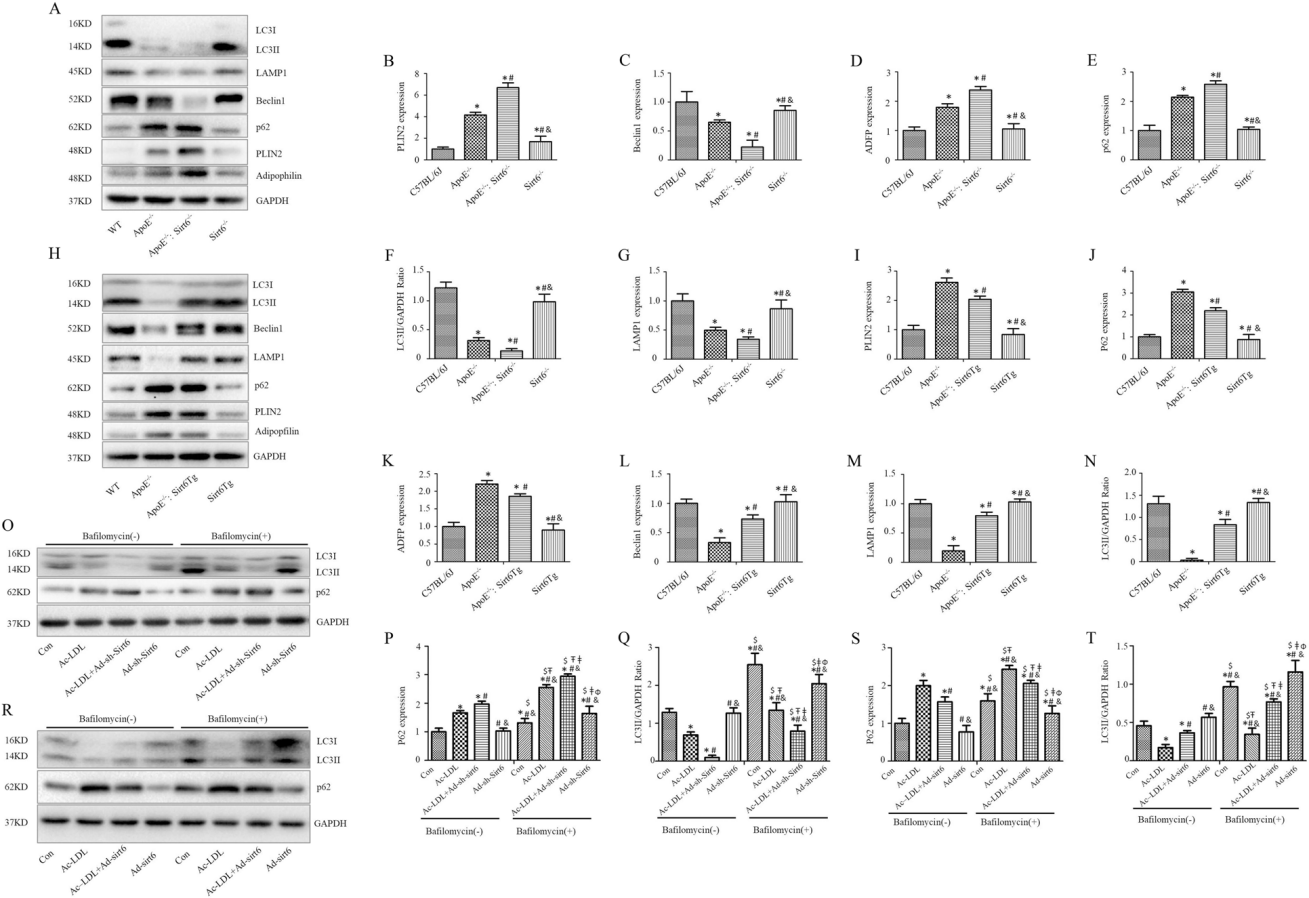
Sirt6 regulates foam cell lipolysis through increased lipophagic flux. **A**–**N** Immunoblots and quantitative analyses of the expression of LC3b, LAMPI, Beclin1, p62, PLIN2 and adipophilin; data are expressed as mean ± SEM (One way ANOVA showed that there was statistical significance among groups. ^*^P < 0.05 versus the C57BL/6J group; ^#^*P* < 0.05 vs the ApoE^−/−^ group; ^&^*P* < 0.05 vs the ApoE^−/−^:Sirt6^−/−^ group [*n* = 10]). **O**–**Q** Results from immunoblotting and quantitative studies of LC3 and p62 in cultured macrophages without or with bafilomycin A1; data are expressed as mean ± SEM (One way ANOVA showed that there was statistical significance among groups. ^*^P < 0.05 vs the control group; ^#^*P* < 0.05 vs the Ac-LDL group; ^&^*P* < 0.05 vs the Ac-LDL + Ad-sh-Sirt6 group; ^$^*P* < 0.05 vs the Ad-sh-Sirt6 group; ^Ŧ^*P* < 0.05 vs the control + bafilomycin A1 group; ^ǂ^*P* < 0.05 vs the Ac-LDL + Ad-sh-Sirt6 + bafilomycin A1 group; ^Ф^*P* < 0.05 vs the Ac-LDL + Ad-Sirt6 + bafilomycin A1 group). **R**–**T** LC3 and p62 levels in cultured macrophages without and with bafilomycin A1 were analyzed by immunoblotting and quantitative analysis (One way ANOVA showed that there was statistical significance among groups. ^*^P < 0.05 vs the control group; ^#^*P* < 0.05 vs the Ac-LDL group; ^&^*P* < 0.05 vs the Ac-LDL + Ad-Sirt6 group; ^$^*P* < 0.05 vs the Ad-Sirt6 group; ^Ŧ^*P* < 0.05 vs the control + bafilomycin A1 group; ^ǂ^*P* < 0.05 vs the Ac-LDL + Ad-Sirt6 + bafilomycin A1 group; ^Ф^*P* < 0.05 vs the Ac-LDL + Ad-Sirt6 + bafilomycin A1 group [*n* = 6 separate experiments])

Adipophilin and PLIN2 have been reported to correlate with atherosclerosis [29–31], their expression was compared among the plaques of ApoE^−/−^, ApoE^−/−^: Sirt6^−/−^ and ApoE^−/−^: Sirt6Tg mice. Compared to those of ApoE^−/−^ mice, enhanced expression of adipophilin and PLIN2 were observed in ApoE^−/−^: Sirt6^−/−^ mice (Fig. 4A–G). While in ApoE^−/−^: Sirt6Tg mice, decreased expression of adipophilin and PLIN2 were found (Fig. 4H–N). Furthermore, the level of LC3, p62 and LAMP1 expression were assessed in plaques to additionally examine the mechanism related to the decreased lipid metabolism in ApoE^−/−^: Sirt6^−/−^ mice. The levels of LC3 and LAMP1 expression were decreased in ApoE^−/−^: Sirt6^−/−^ mice in contrast with that in ApoE^−/−^ mice (Fig. 4A). However, the level of p62 expression was elevated in ApoE^−/−^: Sirt6^−/−^ mice in contrast with that in ApoE^−/−^ mice (Fig. 4A). Simultaneously, ApoE^−/−^: Sirt6Tg mice exhibited increased levels of LC3 and LAMP1 accompanied by decreased p62 expression (Fig. 4H).

In vitro, macrophages treated with 50-ug/mL Ac-LDL following transfection with Ad-sh-Sirt6 led to weakened lipophagic flux along with decreased LC3II expression but increased p62 expression after treatment with bafilomycin A1 (Fig. 4O–Q). However, bafilomycin A1-treated macrophages following transfection with Ad-Sirt6 showed attenuated expression of p62 and enhanced expression of LC3-II (Fig. 4R–T). Altogether, these results indicate that Sirt6 may enhance lipid metabolism by promoting lipophagic flux.

### Sirt6 induces lipid clearance via the autophagy-lysosome degradation pathway

**Fig. 5:**
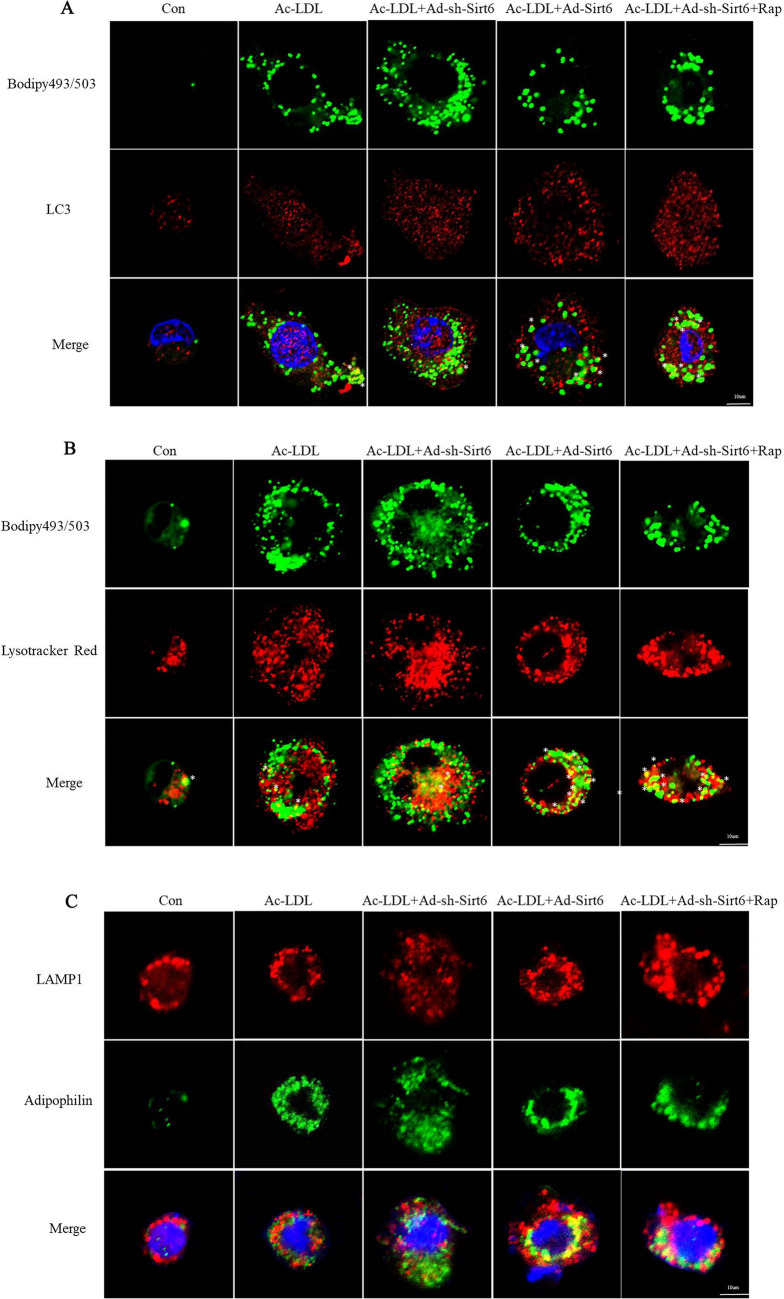
Overexpression of Sirt6 enhances autophagy-mediated breakdown of lipid droplets in macrophages. **A** Co-localisation of LC3 (red) with BODIPY 493/503 (green) in Ac-LDL-treated macrophages cultured in Ad-sh-Sirt6 or Ad-Sirt6 medium. **B** Macrophages were incubated with Ac-LDL for 30 h and subsequently for 30 min with Bodipy 493/503 (10 µg/mL) in the presence or absence of LysoTracker Red (50 nM) before visualisation. **C** Co-localisation between the macrophage lipid droplet coat protein adipophilin (green) and LAMP1-positive lysosomes (red) in Ac-LDL-treated macrophages (*n* = 6 separate experiments)

To verify that autophagy regulates intracellular lipids in macrophages, its effect on LDs degradation was examined. As shown in Fig. 5A-C, transfection with Ad-Sirt6 led to an increased levels of cellular LC3 and lysosome-associated membrane protein 1 (LAMP1) as demonstrated by LysoTracker staining (shown in red); while the expression of adipophilin (shown in green) were decreased. The results of co-staining (shown in yellow) demonstrated a direct association between LDs and autophagosomes (Fig. 5A). In addition, induction of autophagy via Ad-Sirt6 increased the co-localisation of LDs and LC3 (Fig. 5A).

Since lysosomes are the sites of autophagy-induced degradation of macromolecules, including lipids, their hydrolysis effect was examined. The co-localisation of LDs and lysosomes was analysed using LysoTracker Red staining and were observed using confocal microscope (Fig. 5B). Treatment with Ad-Sirt6 increased the number of LDs fused with lysosomes, suggesting that Sirt6 stimulates the degradation of LDs in lysosomes. Furthermore, increased co-localisation of LDs coat protein adipophilin and LAMP1 were observed in macrophages transfected with Ad-Sirt6 (Fig. 5C). Furthermore, to investigate the possible involvement of Sirt6-induced autophagy in the degradation of LDs, macrophages were treated with rapamycin, an autophagy agonist. The result illustrated that treatment with rapamycin promoted the co-localisation of LDs and LC3 in the absence of Sirt6. In addition, the co-localisation of LDs and lysosomes and the co-localisation adipophilin and LAMP1 were also increased in the absence of Sirt6. The above data demonstrate the function of the autophagy-lysosome pathway in the degradation of intracellular lipids mediated by Sirt6.

### Sirt6 stimulates plaque stability by regulating lipophagy

**Fig. 6:**
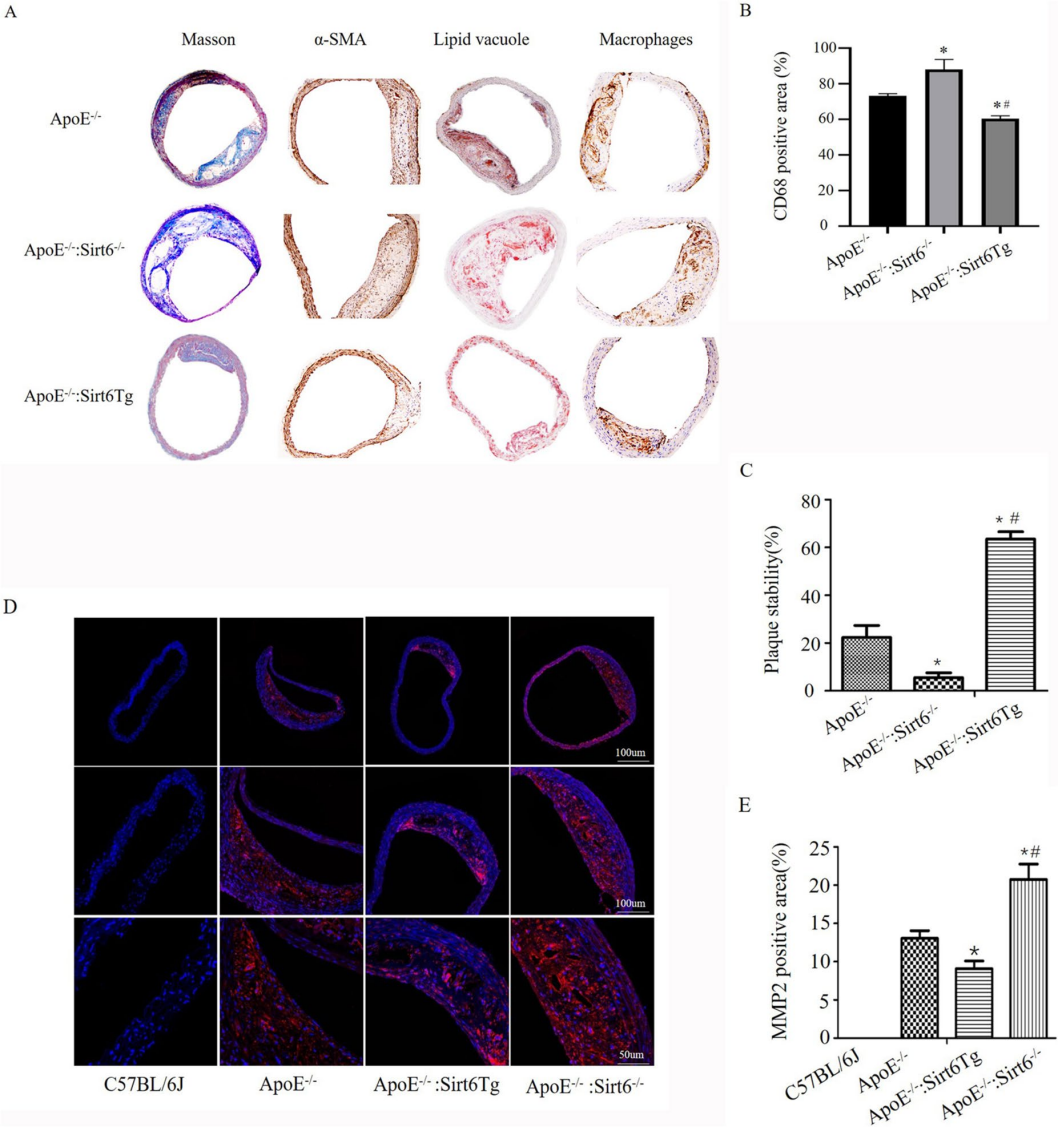
Overexpression of Sirt6 results in enhanced plaque stability. **A** Immunohistochemical analysis of macrophages (CD68) and α-smooth muscle actin (SMA); Collagen was stained using Masson’s reagent, and the lipid vacuole was stained with Oil red O. **B**–**C** Examining CD68 positive area and the plaque stabilisation score quantitatively. Data are presented as mean ± SE (One way ANOVA showed that there was statistical significance among groups, ^*^P < 0.05 vs ApoE^−/−^ mice [*n* = 10]; ^#^*P* < 0.05 vs ApoE^−/−^: Sirt6^−/−^ mice [*n* = 10]). **D** Plaque slices were immunostained with a fluorescent anti-MMP2 antibody. **E** A quantitative study of the plaque area that showed positive staining for MMP2; data are presented as mean ± SE (One way ANOVA showed that there was statistical significance among groups, ^*^P < 0.05 vs ApoE^−/−^ mice [*n* = 10]; ^#^*P* < 0.05 vs ApoE^−/−^: Sirt6Tg [*n* = 10])

During the chronic progression of atherosclerosis, vulnerable plaques are characterized by increased infiltration of macrophages, larger lipid core area, less collagen content and less proportion of vascular smooth muscle cells (VSMCs) [32]. The following equation is derived and used to determine the stability of the plaque. plaque stabilisation score = (α-SMA area [%] + collagen area [%]) / (macrophage area [%] + lipid vacuole area [%]) [33]. In addition, immunohistochemical staining revealed that the proportion of VSMCs and levels of macrophage infiltration were decreased in ApoE^−/−^: Sirt6^−/−^ mice (Fig. 6A-B).

The overexpression of matrix metalloproteinases (MMPs) facilitated the degradation of the fibrous cap’s matrix, leading to plaques instability. In addition, in vulnerable plaques, MMP activity is linked to both the thinner fibrous cap and the larger lipid core, indicating that it might be a useful therapeutic target [34, 35]. In this study, immunofluorescence staining revealed that the MMP2-positive area (Fig. 6C-D) was increased in ApoE^−/−^: Sirt6^−/−^ mice compared with ApoE^−/−^ mice. While ApoE^−/−^: Sirt6Tg mice showed reduced MMP2-positive area compared with ApoE^−/−^ mice and ApoE^−/−^: Sirt6^−/−^ mice. These results suggest that Sirt6 inhibits atherosclerotic progression and promotes plaque stability by regulating lipid metabolism disorder in macrophage foam cells.

### Sirt6 interacts with the chromatin remodeler SNF2H to inhibit Wnt1 signalling, thus promoting lipophagy

**Fig. 7:**
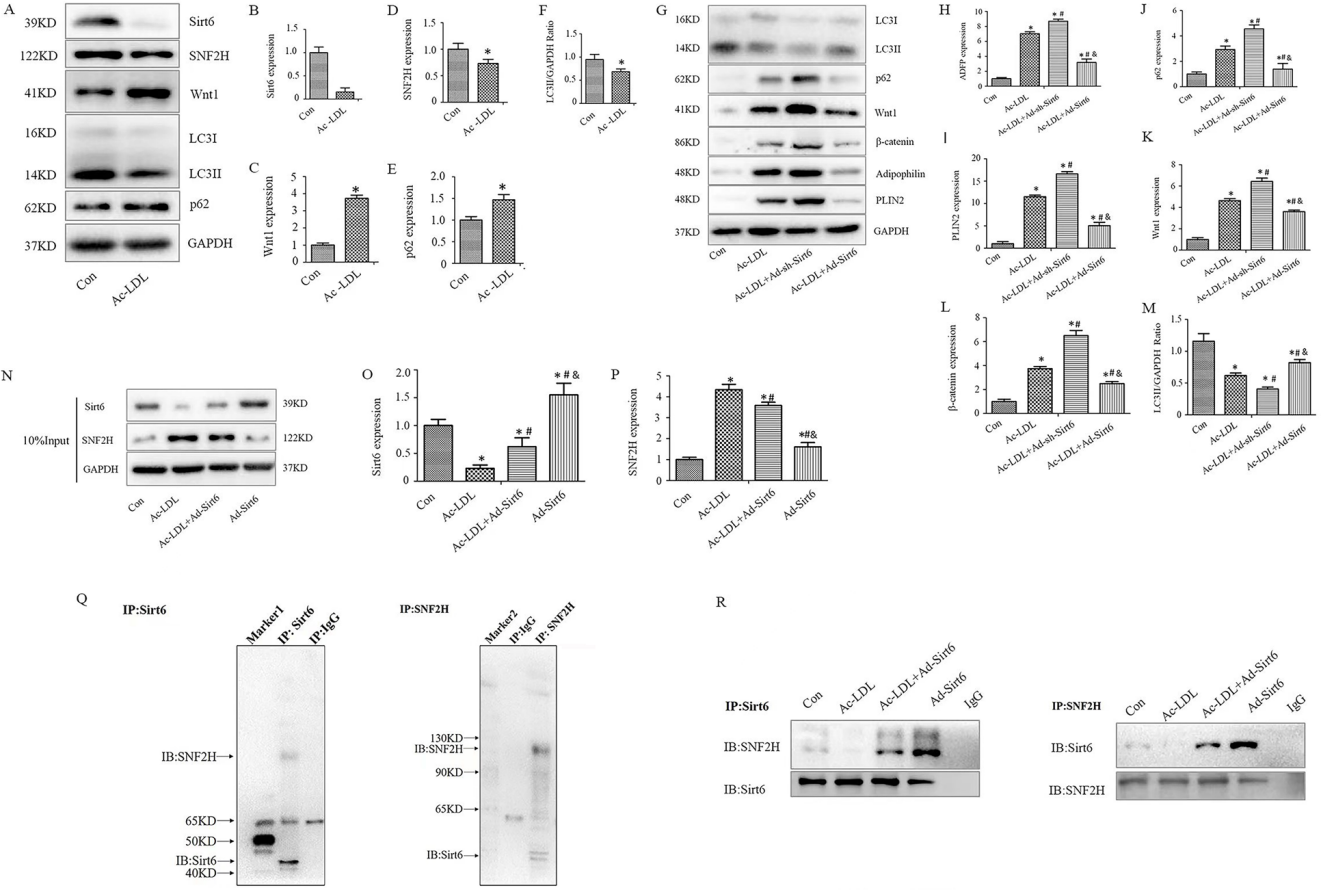
Mechanisms of action of Sirt6 for promoting lipophagy. **A**–**F** Sirt6, SNF2H, Wnt1, LC3-II/GAPDH, and P62 were examined by immunoblotting and quantitative analysis (*n* = 6). **G**–**M** The LC3-II/GAPDH ratio, P62, Wnt1, β-catenin, PLIN2, and adipophilin expression were analyzed quantitatively by immunoblotting (*n* = 6). **N** The 10% input of Co-IP. **O**–**P** Immunoblots and quantitative analyses of the 10% input. **Q**-**R** Co-immunoprecipitation (Co-IP) experiments for Sirt6 and SNF2H. One way ANOVA showed that there was statistical significance among groups (*P* < 0.05)

Wnt signalling has recently been revealed to exert a crucial role in the pathophysiology of atherosclerosis [36]. Activated canonical Wnt/β-catenin pathway promoting monocytes’s adherence to vascular endothelial cells, contributing to foam cells formation [36, 37]. In addition, Wnt1/β-catenin signalling can activate mTOR, leading to a decreased level of autophagy in macrophages [38]. Sirt6 recruits the ISWI-chromatin remodeler SNF2H, which is critical for preventing DNA damage [39]. In this study, western blotting revealed that Ac-LDL-treated macrophages exhibited decreased expression of Sirt6 accompanied by the lowered level of SNF2H and LC3 and the elevated level of Wnt1 and p62 (Fig. 7A–F). Furthermore, transfection with Ad-Sirt6 inhibited the Wnt1/β-catenin signalling pathway and decreased the expression of the LDs-associated proteins ADFP and PLIN2. However, transfection with Ad-sh-Sirt6 activated the Wnt1/β-catenin signalling pathway (Fig. 7G–M). To verify whether Sirt6 regulates lipophagy by inhibiting Wnt1, salinomycin was used to inhibit Wnt1 signalling and found that the regulation of lipophagy by Sirt6 was antagonised by salinomycin (Supplementary Fig. 2A–G). Therefore, Sirt6 may interacted with SNF2H to regulate Wnt1 signalling thus promoting lipophagy was hypothesised in the research.

To verify this hypothesis, co-immunoprecipitation (co-IP) experiments were performed to evaluate the interplay between Sirt6 and SNF2H in macrophages treated with Ac-LDL. Western blotting was used to identify Sirt6 and SNF2H after immunoprecipitation with the SNF2H and Sirt6 antibodies, respectively (Fig. 7O–R). Neither SNF2H nor Sirt6 was detected after the immunoprecipitation of the normal sample. A negative control experiment was performed using control IgG (corresponding to the primary antibody source) using an identical amount of IgG and lysate; however, neither SNF2H nor Sirt6 was detected via western blotting. These results suggested that Sirt6 and SNF2H combined, thereby inactivating Wnt1 signalling, which promoting lipophagy in macrophages treated with Ac-LDL. Furthermore, in order to investigate the function of Sirt6 on macrophage lipid metabolism was SNF2H dependent. The co-localisation of lipid droplets and lysosomes was analysed using LysoTracker (Red) and Bodipy 493/503 (Green) staining and were observed using confocal microscope. Little co-localisation of lipid droplets and lysosomes was observed in the absence of SNF2H while transfected with Ad-Sirt6 in macrophages treated with Ac-LDL (Supplementary Fig. 3). The results indicated that SNF2H was indispensable to Sirt6 that play an important role in regulating lipid metabolic disorders and the formation of foam cells.

## Discussion

Atherosclerosis is widely known that it is caused by lipid dysregulation and aggravated by the formation of plaques in arteries of various sizes [40]. Foam cells, which originate from infiltrating macrophages with excess lipids stored as LDs, exert a critical role in the establishment of vulnerable plaques and the subsequent occurrence of clinical events in patients with atherosclerosis [41]. Foam cell formation is a hallmark of atherosclerosis, but little is known about the mechanisms that might improve lipid metabolism disorder. Our findings illustrated that Sirt6 is implicated in the pathogenesis of atherosclerosis by promoting lipophagy and subsequently improves lipid metabolism disorder in macrophages. This finding is supported by the following: (1) Sirt6 deficiency contributes to macrophage retention and foam cell formation in plaques; (2) Sirt6 modulates foam cell lipolysis through the autophagy–lysosome degradation pathway; (3) Deletion of Sirt6 impairs LDs catabolism in macrophage foam cells; (4) Sirt6 interacts with the chromatin remodeler SNF2H to inhibit Wnt1 signalling, thus promoting lipophagy; (5) Sirt6 stimulates plaque stability by regulating lipophagy. These results identify Sirt6 as a potential therapeutic target for atherosclerotic plaques.

Atherosclerotic plaque instability is caused by a variety of factors, in which macrophage foam cells are the primary contributor [42]. Macrophages uptake lipoproteins and the clearance of lipids from macrophage cells determine the formation of foam cells [5]. In atherosclerotic plaque, foam cells secret substances such MMPs, nitric oxide (NO), and endothelin, all of which contribute to the plaque’s instability [43, 44]. Atherosclerotic plaques have abnormal macrophage lipid metabolism [45]. Macrophages with overloaded lipids block themselves to emigrate from plaques, resulting in apoptosis and necrosis, which, in turn, makes the plaques even more unstable [41, 42]. Patients with stable atherosclerotic plaques may experience improved clinical outcomes by addressing poor lipid metabolism in foam cells and stimulating the migration of macrophages from the plaques. Therefore, strategies aimed at improving lipid metabolism disorders in macrophage foam cells may help to treat unstable plaques [46]. This investigation found that Sirt6 regulates foam cell formation and promotes lipid degradation in foam cells; hence, it is a unique modulator of macrophage retention. In addition, Sirt6 may serve as a viable target for regulating dysfunctional lipid metabolism in macrophages, thus promoting plaque stability.

Early studies have demonstrated that lesional macrophages absorb large amounts of cholesterol, changing into “foam cells” that are filled with cholesterol ester droplets [47]. LDs are specialised organelles that develop from the endoplasmic reticulum membrane and have a lipid bilayer composed of structural proteins like perilipin [48]. LDs are found most abundantly in macrophages and other cell types such as hepatocytes and neurons [49–51]. PLIN2 has been described as the only constitutive LDs protein, and the level of PLIN2 are correlated with LDs density [30, 31]. Adipophilin is an LDs coat protein found on macrophages, and its abundance is linked to the content of neutral lipids in cells and the risk of developing atherosclerosis [29]. The intracellular storage and utilisation of lipids are essential for maintaining cellular energy homeostasis [18]. Despite this, the processes that reducing foam cell formation and promoting LDs degradation are not fully grasped. This study sheds light on a previously unknown mechanism through which lipid metabolic disorders contribute to macrophage foam cells formation. Sirt6 was shown to function as a unique modulator of Wnt1 inhibition in macrophages treated with Ac-LDL. Some research reports have illustrated the involvement of Wnt signalling in many aspects of the progression of an atherosclerotic lesion, such as macrophage differentiation and infiltration [36]. Wnt1 has been shown to activate mTOR, a central negative regulator of autophagy [38]. A recent study demonstrated that Sirt6 inhibits Wnt signalling by deacetylating histone 3 at lysine 56, which is critical for hematopoietic stem cell (HSC) homeostasis [38]. Until recently, the relationship between SNF2H and atherosclerosis remained unclear. Consistent with previous studies, this study revealed that the effects of Sirt6-induced Wnt1 inhibition are also SNF2H-dependent. In addition, Sirt6 interacts with SNF2H as a novel regulator during atherosclerosis, inhibits Wnt1/β-catenin signalling and promotes lipid degradation. The recently discovered interplay between Sirt6 and Wnt1 promotes lipid degradation and suppresses foam cell formation in plaques.

Lipophagy refers to the metabolic process of lipids. TGs and cholesterol that are stored in LDs are consumed by autophagosomes and subsequently transferred to lysosomes, where they are degraded by acidic hydrolases, eventually releasing FAs and free cholesterol into the cytoplasm [18, 52]. However, the role and mechanisms of lipophagy during atherosclerosis remain unknown. RCT has been reported to reverse plaque lipid build-up in macrophages, which is a process that increases the rate at which cholesterol is transported from the peripheral tissues to the liver, where it is excreted [53]. Mireille Ouimet observed that lipophagy is implicated in the mobilisation of LD-related cholesterol for RCT via the autophagy–lysosome degradation pathway [19]. Previous research has shown that autophagy serves a fundamental function in lipid metabolism, which is linked to a wide variety of human metabolic illnesses including metabolic syndrome [19]. Yet, neither the involvement of lipophagy in atherosclerosis nor its underlying processes have been conclusively established. In this study, we demonstrated a previously unknown inter-relationship between Sirt6-mediated autophagy and lipid metabolism using WT, ApoE^−/−^, ApoE^−/−^ + Rap and ApoE^−/−^: ATG5^flox/flox^Lyz2-cre mice fed a western diet. Furthermore, autophagy was verified that it can reduce the lipid areas in plaques, and Sirt6 can control the expression of adipophilin and PLIN2 under Ac-LDL stimulation. These findings indicate that macrophagic Sirt6 functions as an upstream modulator in conjunction with Wnt1 to induce the lipophagy that promotes the degradation of LDs in foam cells, thus delaying the progression of atherosclerosis.

Furthermore, this study revealed that Sirt6 regulates lipid metabolism mainly by inhibiting Wnt1 in plaques, and one of the signalling molecules downstream of Sirt6 in macrophages is Wnt1/β-catenin. Simultaneously, the impact of Sirt6 on Wnt1 inactivation in response to Ac-LDL was found to be SNF2H-dependent. However, SNF2H-independent pathways responsible for Wnt1 inactivation may exist. Both Netrin-1 and CCR-7 play a role in the retention of macrophages [4]. However, further investigation is required to assess whether Sirt6 regulates the expression of netrin-1 and CCR-7 and whether SNF2H-independent pathways are implicated in the alleviation of atherosclerotic plaques under Sirt6-deficient conditions. Further studies are warranted to identify additional mechanisms of action of Sirt6 in the modulation of FA flux into mitochondria and the underlying mechanisms of Sirt6-mediated lipophagy in regulating mitochondrial dynamics, thereby making this pathway a putative treatment target for atherosclerosis. These aspects will be considered in our future studies.

### Study strengths and limitations

The study has numerous strengths. This investigation focused on macrophage lipid metabolism, exploring the molecular mechanisms that Sirt6 mediated lipophagy promotes lipid metabolism in macrophages to inhibit foam cell formation. Furthermore, this study showed the potential role of Sirt6 mediated lipophagy in increasing atherosclerotic plaque stability, and determined that the Sirt6/Wnt1/β-catenin pathway is a promising therapeutic target.

Sirt6 is a key stimulator of atherosclerotic plaque stabilisation and acts by promoting autophagy, as shown in the previous study. In addition, Sirt6 could help to promote lipophagy to improve dysregulated lipid metabolism in macrophages, thus preventing foam cell formation. In any case, future investigations are required to verify whether Sirt6 regulates FAs flux into mitochondria and its corresponding mechanisms and the level of Sirt6 expression in VSMCs in ApoE^−/−^ mice are warranted to evaluate.

## Conclusions

In summary, Sirt6-mediated autophagy performs a fundamental function in lipid metabolism and subsequent LDs clearance in macrophage foam cells. Sirt6 enhanced autophagy-dependent macrophage lipid metabolism both in vivo and in vitro. Furthermore, autophagy-specific Sirt6 deficiency was found to be detrimental to lipid degradation, and impairment of macrophage autophagy induced by Sirt6 deficiency promoted atherosclerotic lipid accumulation. This study provides a theoretical basis for preventing the development of atherosclerosis in patients with coronary heart disease, improving the stability of plaques, thus reducing the incidence of acute myocardial infarction.

### Supplementary Information

**Additional file 1.**

**Additional file 2: Supplementary Figure 1.** siRNA ATG5 and siRNA ATG7 worked by western blot. (A, C) Expression of ATG5 and ATG7 treated with siRNA ATG5 and siRNA ATG7 were evaluated by western blot; (B, D) Quantitative analysis of ATG5 and ATG7 expression by western blot. Data are expressed as mean ± SE (Student’s t-test showed that there was statistical significance between Con group and siRNA ATG5 with siRNA ATG7 group. * *P* < 0.01 vs the control group [*n* = 6]).

**Additional file 3: Supplementary Figure 2.** Sirt6 regulates lipophagy by inhibiting Wnt1. (A–G) Analysis of LC3, Beclin1, LAMP1, adipophilin, PLIN2, and P62 by immunoblotting and quantitative analysis (*n* = 6). Data are expressed as mean ± SE (One way ANOVA showed that there was statistical significance among groups. ^*^ *P* < 0.05 vs the control group; ^#^*P* < 0.05 vs the ox-LDL group; ^&^*P* < 0.05 vs the Ac-LDL+salinomycin group; ^$^*P* < 0.05 vs the ox-LDL+Ad-Sirt6 group [*n* = 6]).

**Additional file 4: Supplementary Figure 3.** The effect of Sirt6 on lipid droplets degradation was SNF2H dependent. (A) Co-localization of lipid droplets and lysosomes analyzed by LysoTracker Red and BODIPY 493/503 staining were detected by confocal microscopy in the presence or absence of SNF2H administration (*n*=6 separate experiments).

## Data Availability

This article incorporates all data generated or evaluated throughout this investigation.

## Abbreviations

Ac-LDL: Acetylated low-density lipoprotein
ASCVD: Atherosclerotic cardiovascular disease
LDL-C: Low-density lipoprotein cholesterol
TGs: Postprandial triglycerides
Ox-LDL: Oxidised LDL
VLDL: Very-low-density lipoprotein
RCT: Reverse cholesterol transport
HDL: High-density lipoprotein
LDs: Lipid droplets
ORO: Oil red O staining
DAPI: 4,6-Diamidino-2-phenylindole
LAMP1: Lysosome-associated membrane protein 1
FAs: Fatty acids
