# Supplementary material for: Sirt6 enhances macrophage lipophagy and improves lipid metabolism disorder by regulating the Wnt1/β-catenin pathway in atherosclerosis
Source: Lipids Health Dis. 2023 Sep 22;22:156. doi: 10.1186/s12944-023-01891-3 (PMC10515036; doi:10.1186/s12944-023-01891-3)
Supplement: Supplementary file 1 — Additional file 1. [file 12944_2023_1891_MOESM1_ESM.zip › Supplemental Materials/Editorial certificate.pdf]

Bullet Edits

EDITORIAL CERTIFICATE

This document certifies that the paper listed below was edited and proofread for proper English language, grammar, punctuation, spelling, and overall style by one or more than one highly qualified native speakers at Bullet Edits. All of the suggested amendments were tracked with the Microsoft Word "Track Changes" feature. Therefore, the author had the option to reject or accept each change individually.

Manuscript Title:

Sirt6 enhances macrophage lipophagy and improves lipid metabolism disorder by regulating the Wnt1/β-catenin pathway in atherosclerosis

Anti-counterfeiting Code:

561943f4ed1da28b4e7dc8e0693f8e85

Date Issued:

2022-05-24 19:41:08

Bullet Edits is a registered company headquartered in the UK with a global presence. We offer a range of editing, proofreading services to authors. Our Ph.D. editors are all native English speakers from the USA and UK. Authors who work with Bullet Edits are guaranteed excellent language quality and timely delivery.

BULLET EDITS LIMITED ★ COMPANY NO. 13345261 SEAL ★ ENGLAND AND WALES

Address: BULLET EDITS LIMITED, 85 Great Portland Street, London,UK
Tel: UK. (+44)20457 70286 / US.(+1)312-313-9179
Web: www.bulletedits.cn/Email: info@bulletedits.cn
VAT: GB 378 9316 43
