# Supplementary material for: Sirt6 enhances macrophage lipophagy and improves lipid metabolism disorder by regulating the Wnt1/β-catenin pathway in atherosclerosis
Source: Lipids Health Dis. 2023 Sep 22;22:156. doi: 10.1186/s12944-023-01891-3 (PMC10515036; doi:10.1186/s12944-023-01891-3)
Supplement: Supplementary file 1 — Additional file 1. [file 12944_2023_1891_MOESM1_ESM.zip › Supplemental Materials/Supplemental Material Methods.docx]

Supplemental Material

**Detailed Methods**

**Cell Preparation**

Pre-cool a refrigerated centrifuge to 4℃. Count total cells present in the sample before centrifugation using a hemocytometer. Pellet cultured cells by centrifugation for 5 minutes at 500$\times$g(approximately 2100 rpm for rotors of 9.5 cm radius) at 4°C. Wash 3 times with ice-cold 1$\times$PBS and then add chilled lysis buffer with 1$\times$protease inhibitor added. Add 100ul IP lysis buffer for approximately every 10^6^ cells present in the pellet. Reduce the volume of IP lysis buffer accordingly if a higher protein concentration is required. Add phosphatase inhibitors to IP lysis buffer for extraction of phosphorylated proteins. Resuspend cells in the IP lysis buffer by vortexing briefly. Keep the mixture on ice for 30 minutes, vortexing occasionally. See lysis and storage for additional steps.

**Preparing protein A sepharose beads**

Resuspend protein A sepharose beads in the bead slurry gy gently vortexing the storage bottle. Spin briefly and wash bead pellet 3-5 times with rotally ten times the pellet volume using chilled 1$\times$PBS.

**Pre-clearing the lysate(optional)**

Shear off the end of a sterile pipette tip at an angle of 45°（making sure it still retains suction）and use the modified tip to quickly add 50ul of 50% protein A sepharose beads slurry to the microfuge tube containing your lysate. In general add 30ul of resuspended beads per 1-3mg of total protein. Adjust the volume of resuspended beads accordingly. Incubate on a rotary mixer for 60 minutes at 4℃. Centrifuge at 1000rpm for 1 minutes at 4℃ and transfer the aupernatant to a fresh tube.

**Immunoprecipitation**

1.Transfer 200-350ul of your whole(or precleared) lysate containing 1-3mg of total protein to a spin column with end caps in place. Then add 1-4ug of primary antibody(Sirt6) and 150-300ul incubation buffer. Optimal antibody amount should be determined by titration. Set up a negative control experiment with control IgG (corresponding to the primary antibody source) using identical amount of IgG and lysate (the following operation is exactly the same as the target sample tube). Gently rock the incubation at 4℃ for 2-4 hours or overnight. Add 50ul resuspended protein A sepharose beads to the spin columns to capture the immunocomplex. Gently rock the mixture at 4℃ for 1-4 hours. Take off the end caps, the supernatant is released from the spin columns bottom naturally. If necessary, resuspend the beads mixture to enhance the flow velocity. Wash the beads 4-5 times with 1$\times$washing buffer(800ul each time) containing 1$\times$protease inhibitor. If necessary, centrifuge the apin columns at 500rpm for 30 seconds at 4℃ and collect the supernatant with collection tubes and discard it.

2.Transfer 200-350ul of your whole(or precleared) lysate containing 1-3mg of total protein to a spin column with end caps in place. Then add 1-4ug of primary antibody(SNF-2H) and 150-300ul incubation buffer. Optimal antibody amount should be determined by titration. Set up a negative control experiment with control IgG (corresponding to the primary antibody source) using identical amount of IgG and lysate (the following operation is exactly the same as the target sample tube). Gently rock the incubation at 4℃ for 2-4 hours or overnight. Add 50ul resuspended protein A sepharose beads to the spin columns to capture the immunocomplex. Gently rock the mixture at 4℃ for 1-4 hours. Take off the end caps, the supernatant is released from the spin columns bottom naturally. If necessary, resuspend the beads mixture to enhance the flow velocity. Wash the beads 4-5 times with 1$\times$washing buffer(800ul each time) containing 1$\times$protease inhibitor. If necessary, centrifuge the apin columns at 500rpm for 30 seconds at 4℃ and collect the supernatant with collection tubes and discard it.

**Elution**

Place spin columns in a fresh microfuge tube and pool the elutions. Elute the pellet with 40ul elution buffer and centrifuge at 10000 rpm for 1 min at 4℃, once again with new 40ul elution buffer. Add 10ul alkali neutralization buffer and 23ul 5$\times$sample buffer to all the elutions, heat at 95℃ for 5min.

**Finally Western blotting analysis**

Probe with appropriate antibodies and HRP-conjugated mouse anti-rabbit IgG light chain specific at 1:1000-1:2000 titer.
