# Supplementary figures and images for: Sirt6 enhances macrophage lipophagy and improves lipid metabolism disorder by regulating the Wnt1/β-catenin pathway in atherosclerosis
Source: Lipids Health Dis. 2023 Sep 22;22:156. doi: 10.1186/s12944-023-01891-3 (PMC10515036; doi:10.1186/s12944-023-01891-3)

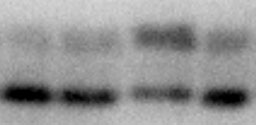

Supplement: Supplementary file 1 — Additional file 1. [file 12944_2023_1891_MOESM1_ESM.zip › Supplemental Materials/original blots/Fig4A/Fig 4A LC3-2.jpg]

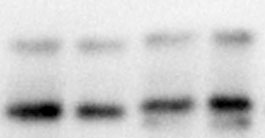

Supplement: Supplementary file 1 — Additional file 1. [file 12944_2023_1891_MOESM1_ESM.zip › Supplemental Materials/original blots/Fig4A/Fig 4A LC3-3.jpg]

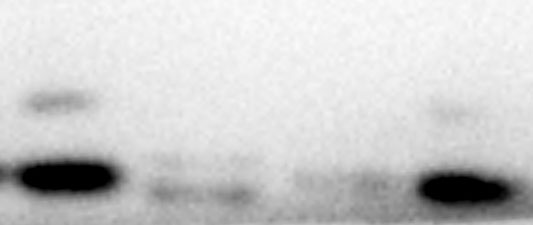

Supplement: Supplementary file 1 — Additional file 1. [file 12944_2023_1891_MOESM1_ESM.zip › Supplemental Materials/original blots/Fig4A/Fig 4A LC3.jpg]

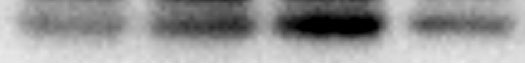

Supplement: Supplementary file 1 — Additional file 1. [file 12944_2023_1891_MOESM1_ESM.zip › Supplemental Materials/original blots/Fig4A/Fig4A Adipophilin-1.jpg]

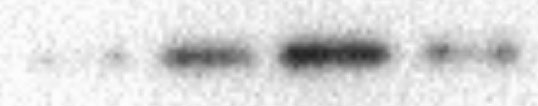

Supplement: Supplementary file 1 — Additional file 1. [file 12944_2023_1891_MOESM1_ESM.zip › Supplemental Materials/original blots/Fig4A/Fig4A Adipophilin-2.jpg]

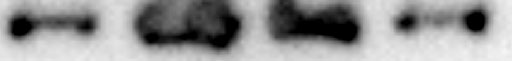

Supplement: Supplementary file 1 — Additional file 1. [file 12944_2023_1891_MOESM1_ESM.zip › Supplemental Materials/original blots/Fig4A/Fig4A Adipophilin-3.jpg]

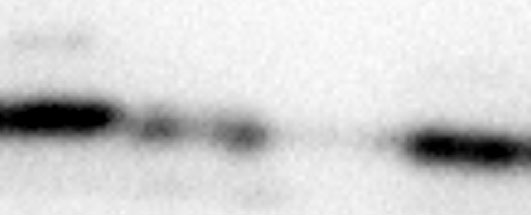

Supplement: Supplementary file 1 — Additional file 1. [file 12944_2023_1891_MOESM1_ESM.zip › Supplemental Materials/original blots/Fig4A/Fig4A Beclin1-1.jpg]

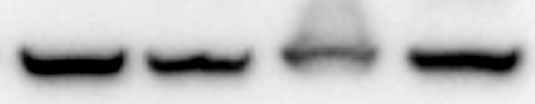

Supplement: Supplementary file 1 — Additional file 1. [file 12944_2023_1891_MOESM1_ESM.zip › Supplemental Materials/original blots/Fig4A/Fig4A Beclin1-3.jpg]

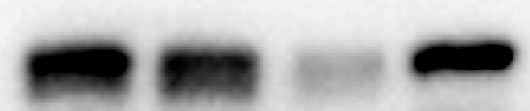

Supplement: Supplementary file 1 — Additional file 1. [file 12944_2023_1891_MOESM1_ESM.zip › Supplemental Materials/original blots/Fig4A/Fig4A Beclin1.jpg]

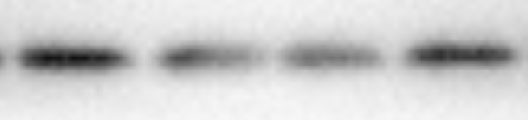

Supplement: Supplementary file 1 — Additional file 1. [file 12944_2023_1891_MOESM1_ESM.zip › Supplemental Materials/original blots/Fig4A/Fig4A LAMP-1.jpg]

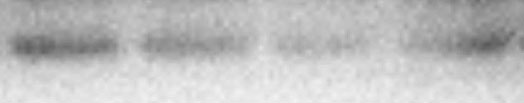

Supplement: Supplementary file 1 — Additional file 1. [file 12944_2023_1891_MOESM1_ESM.zip › Supplemental Materials/original blots/Fig4A/Fig4A LAMPI-2.jpg]

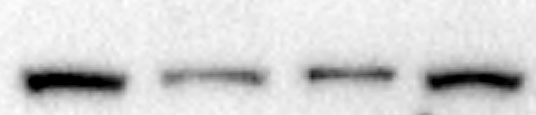

Supplement: Supplementary file 1 — Additional file 1. [file 12944_2023_1891_MOESM1_ESM.zip › Supplemental Materials/original blots/Fig4A/Fig4A LAMPI-3.jpg]

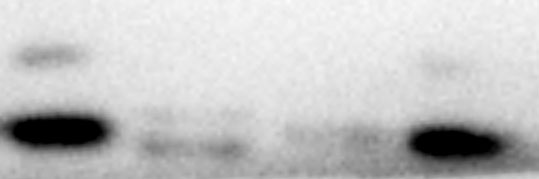

Supplement: Supplementary file 1 — Additional file 1. [file 12944_2023_1891_MOESM1_ESM.zip › Supplemental Materials/original blots/Fig4A/Fig4A LC3-1.jpg]

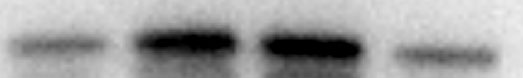

Supplement: Supplementary file 1 — Additional file 1. [file 12944_2023_1891_MOESM1_ESM.zip › Supplemental Materials/original blots/Fig4A/Fig4A P62-1.jpg]

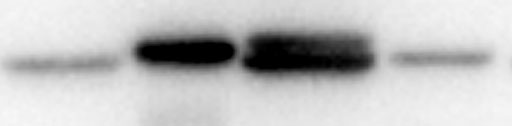

Supplement: Supplementary file 1 — Additional file 1. [file 12944_2023_1891_MOESM1_ESM.zip › Supplemental Materials/original blots/Fig4A/Fig4A P62-2.jpg]

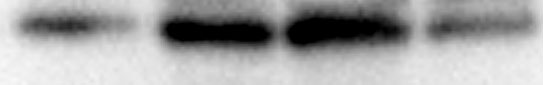

Supplement: Supplementary file 1 — Additional file 1. [file 12944_2023_1891_MOESM1_ESM.zip › Supplemental Materials/original blots/Fig4A/Fig4A P62-3.jpg]

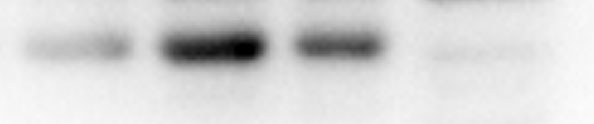

Supplement: Supplementary file 1 — Additional file 1. [file 12944_2023_1891_MOESM1_ESM.zip › Supplemental Materials/original blots/Fig4A/Fig4A PLIN2-1.jpg]

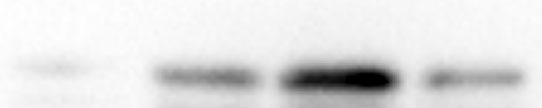

Supplement: Supplementary file 1 — Additional file 1. [file 12944_2023_1891_MOESM1_ESM.zip › Supplemental Materials/original blots/Fig4A/Fig4A PLIN2-2.jpg]

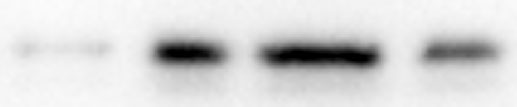

Supplement: Supplementary file 1 — Additional file 1. [file 12944_2023_1891_MOESM1_ESM.zip › Supplemental Materials/original blots/Fig4A/Fig4A PLIN2-3.jpg]

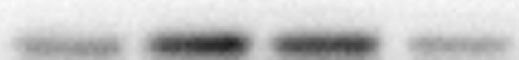

Supplement: Supplementary file 1 — Additional file 1. [file 12944_2023_1891_MOESM1_ESM.zip › Supplemental Materials/original blots/Fig4H/Fig4H Adipopfilin-1.jpg]

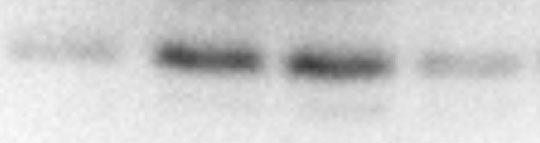

Supplement: Supplementary file 1 — Additional file 1. [file 12944_2023_1891_MOESM1_ESM.zip › Supplemental Materials/original blots/Fig4H/Fig4H Adipopfilin-2.jpg]

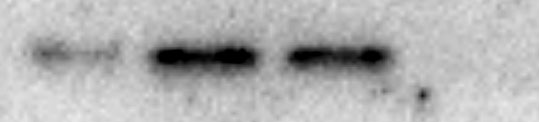

Supplement: Supplementary file 1 — Additional file 1. [file 12944_2023_1891_MOESM1_ESM.zip › Supplemental Materials/original blots/Fig4H/Fig4H Adipopfilin-3.jpg]

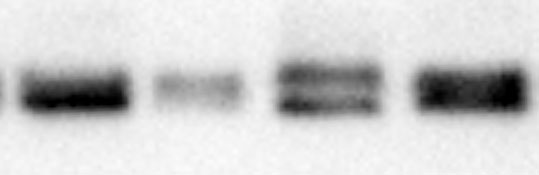

Supplement: Supplementary file 1 — Additional file 1. [file 12944_2023_1891_MOESM1_ESM.zip › Supplemental Materials/original blots/Fig4H/Fig4H Beclin1-1.jpg]

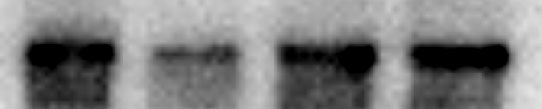

Supplement: Supplementary file 1 — Additional file 1. [file 12944_2023_1891_MOESM1_ESM.zip › Supplemental Materials/original blots/Fig4H/Fig4H Beclin1-3.jpg]

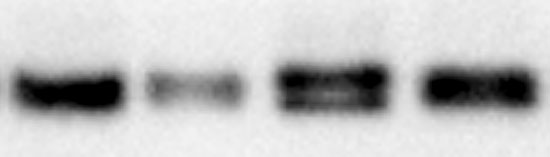

Supplement: Supplementary file 1 — Additional file 1. [file 12944_2023_1891_MOESM1_ESM.zip › Supplemental Materials/original blots/Fig4H/Fig4H Beclin1.jpg]

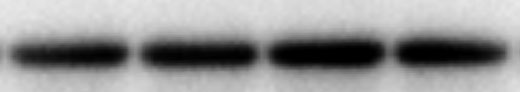

Supplement: Supplementary file 1 — Additional file 1. [file 12944_2023_1891_MOESM1_ESM.zip › Supplemental Materials/original blots/Fig4H/Fig4H GAPDH.jpg]

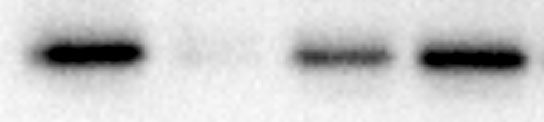

Supplement: Supplementary file 1 — Additional file 1. [file 12944_2023_1891_MOESM1_ESM.zip › Supplemental Materials/original blots/Fig4H/Fig4H LAMPI-2.jpg]

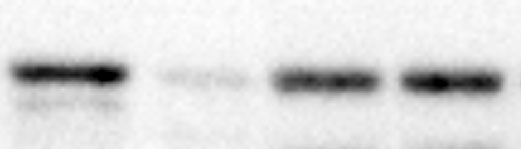

Supplement: Supplementary file 1 — Additional file 1. [file 12944_2023_1891_MOESM1_ESM.zip › Supplemental Materials/original blots/Fig4H/Fig4H LAMPI-3.jpg]

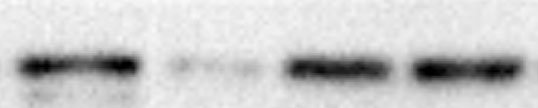

Supplement: Supplementary file 1 — Additional file 1. [file 12944_2023_1891_MOESM1_ESM.zip › Supplemental Materials/original blots/Fig4H/Fig4H LAMPI.jpg]

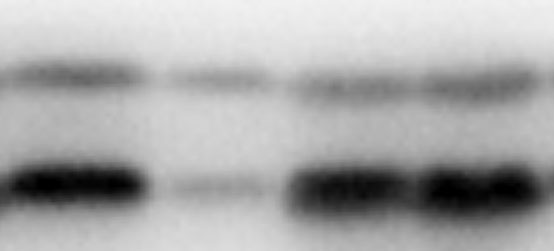

Supplement: Supplementary file 1 — Additional file 1. [file 12944_2023_1891_MOESM1_ESM.zip › Supplemental Materials/original blots/Fig4H/Fig4H LC3-1.jpg]

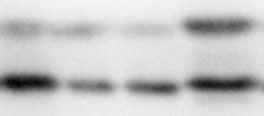

Supplement: Supplementary file 1 — Additional file 1. [file 12944_2023_1891_MOESM1_ESM.zip › Supplemental Materials/original blots/Fig4H/Fig4H LC3-2.jpg]

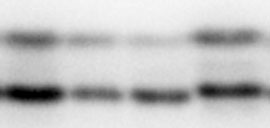

Supplement: Supplementary file 1 — Additional file 1. [file 12944_2023_1891_MOESM1_ESM.zip › Supplemental Materials/original blots/Fig4H/Fig4H LC3-3.jpg]

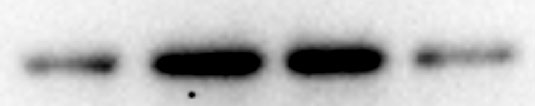

Supplement: Supplementary file 1 — Additional file 1. [file 12944_2023_1891_MOESM1_ESM.zip › Supplemental Materials/original blots/Fig4H/Fig4H P62-1.jpg]

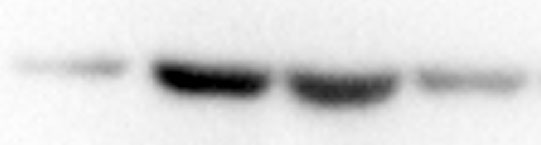

Supplement: Supplementary file 1 — Additional file 1. [file 12944_2023_1891_MOESM1_ESM.zip › Supplemental Materials/original blots/Fig4H/Fig4H P62-2.jpg]

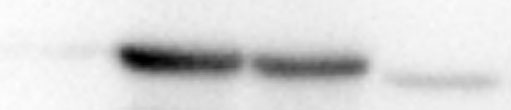

Supplement: Supplementary file 1 — Additional file 1. [file 12944_2023_1891_MOESM1_ESM.zip › Supplemental Materials/original blots/Fig4H/Fig4H P62-3.jpg]

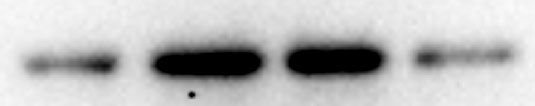

Supplement: Supplementary file 1 — Additional file 1. [file 12944_2023_1891_MOESM1_ESM.zip › Supplemental Materials/original blots/Fig4H/Fig4H P62.jpg]

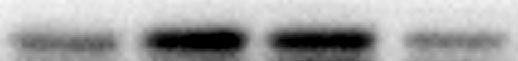

Supplement: Supplementary file 1 — Additional file 1. [file 12944_2023_1891_MOESM1_ESM.zip › Supplemental Materials/original blots/Fig4H/Fig4H PLIN2-1.jpg]

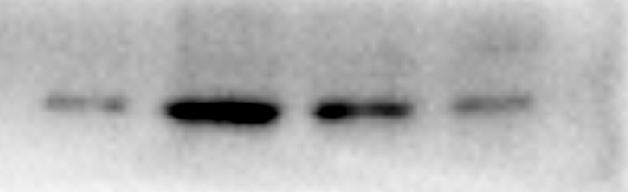

Supplement: Supplementary file 1 — Additional file 1. [file 12944_2023_1891_MOESM1_ESM.zip › Supplemental Materials/original blots/Fig4H/Fig4H PLIN2-2.jpg]

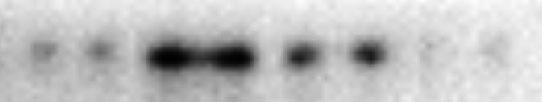

Supplement: Supplementary file 1 — Additional file 1. [file 12944_2023_1891_MOESM1_ESM.zip › Supplemental Materials/original blots/Fig4H/Fig4H PLIN2.jpg]

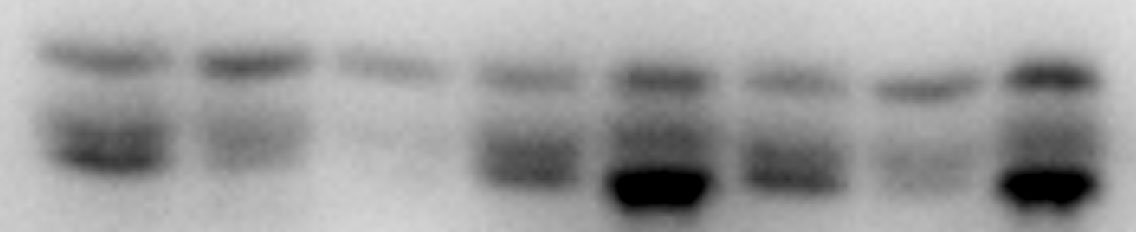

Supplement: Supplementary file 1 — Additional file 1. [file 12944_2023_1891_MOESM1_ESM.zip › Supplemental Materials/original blots/Fig4O/Fig4 O LC3-1.jpg]

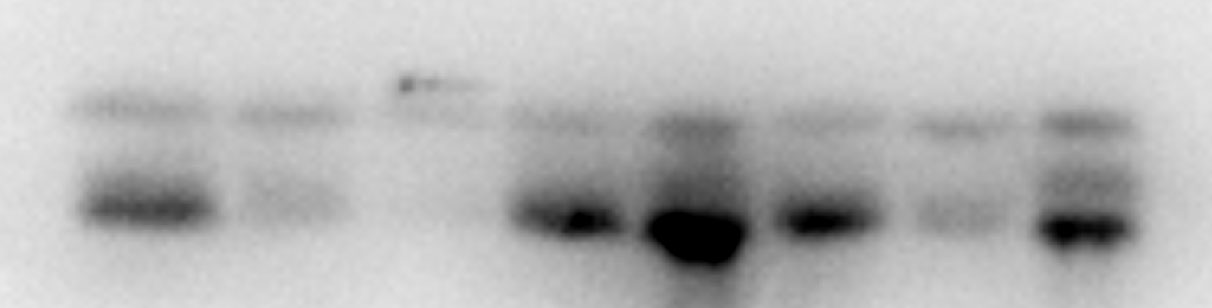

Supplement: Supplementary file 1 — Additional file 1. [file 12944_2023_1891_MOESM1_ESM.zip › Supplemental Materials/original blots/Fig4O/Fig4O LC3-2.jpg]

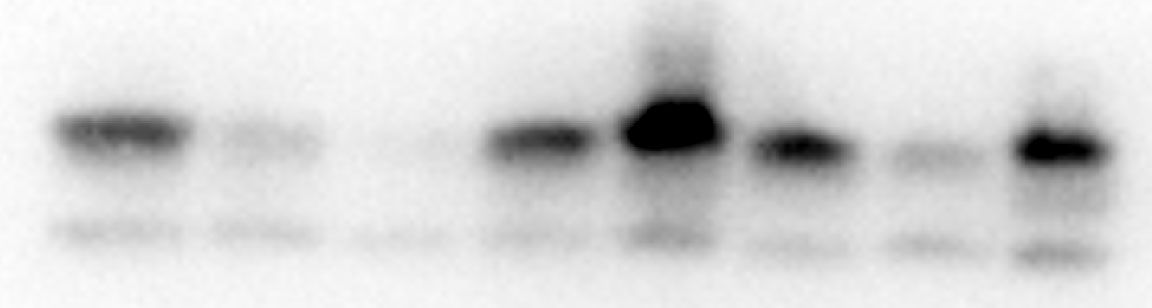

Supplement: Supplementary file 1 — Additional file 1. [file 12944_2023_1891_MOESM1_ESM.zip › Supplemental Materials/original blots/Fig4O/Fig4O LC3-3.jpg]

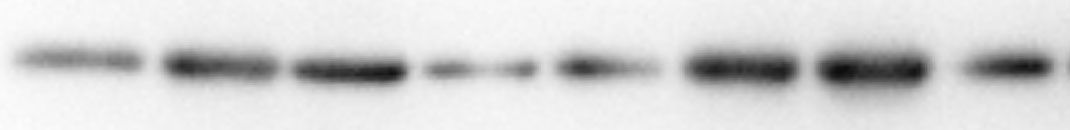

Supplement: Supplementary file 1 — Additional file 1. [file 12944_2023_1891_MOESM1_ESM.zip › Supplemental Materials/original blots/Fig4O/Fig4O P62-1.jpg]

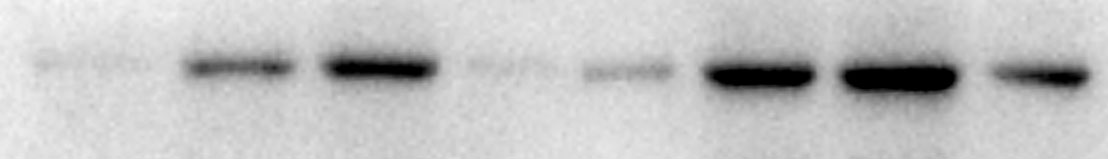

Supplement: Supplementary file 1 — Additional file 1. [file 12944_2023_1891_MOESM1_ESM.zip › Supplemental Materials/original blots/Fig4O/Fig4O P62-3.jpg]

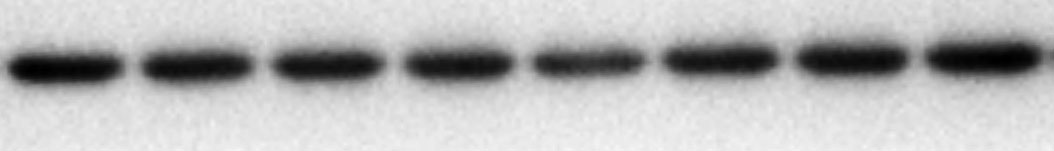

Supplement: Supplementary file 1 — Additional file 1. [file 12944_2023_1891_MOESM1_ESM.zip › Supplemental Materials/original blots/Fig4O/GAPDH.jpg]

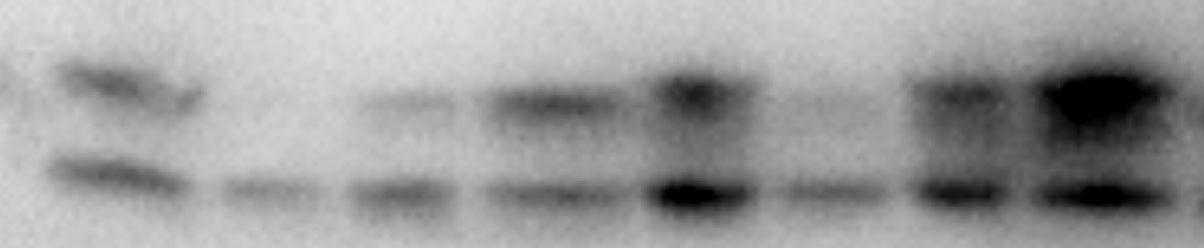

Supplement: Supplementary file 1 — Additional file 1. [file 12944_2023_1891_MOESM1_ESM.zip › Supplemental Materials/original blots/Fig4R/Fig4R LC3-1.jpg]

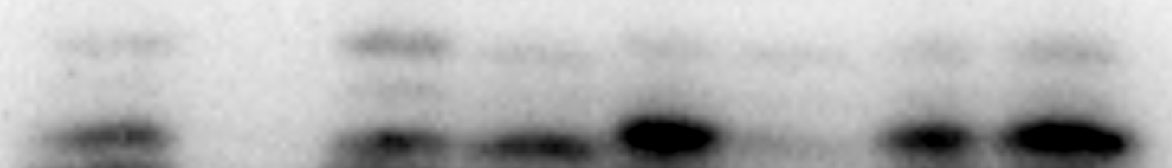

Supplement: Supplementary file 1 — Additional file 1. [file 12944_2023_1891_MOESM1_ESM.zip › Supplemental Materials/original blots/Fig4R/Fig4R LC3-2.jpg]

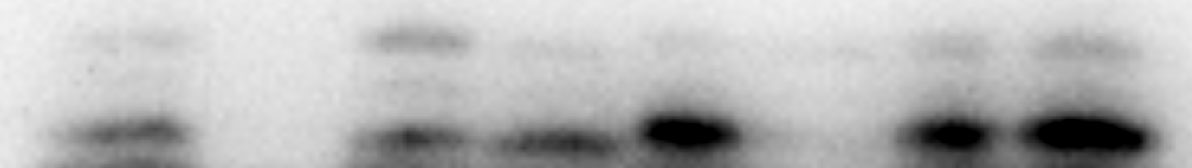

Supplement: Supplementary file 1 — Additional file 1. [file 12944_2023_1891_MOESM1_ESM.zip › Supplemental Materials/original blots/Fig4R/Fig4R LC3-3.jpg]

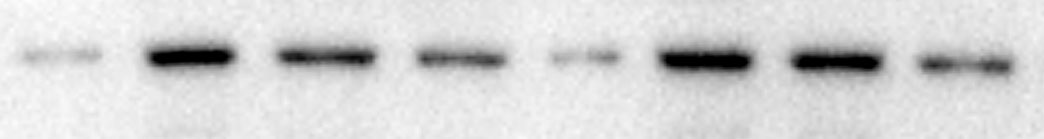

Supplement: Supplementary file 1 — Additional file 1. [file 12944_2023_1891_MOESM1_ESM.zip › Supplemental Materials/original blots/Fig4R/Fig4R P62-1.jpg]

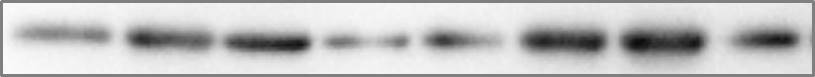

Supplement: Supplementary file 1 — Additional file 1. [file 12944_2023_1891_MOESM1_ESM.zip › Supplemental Materials/original blots/Fig4R/Fig4R P62-2.jpg]

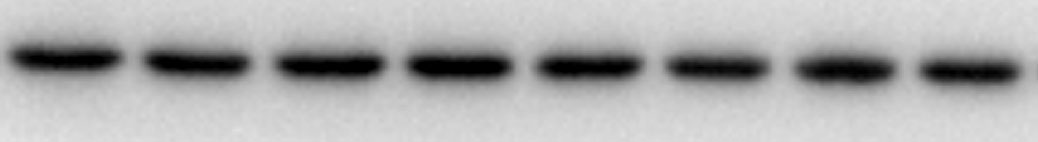

Supplement: Supplementary file 1 — Additional file 1. [file 12944_2023_1891_MOESM1_ESM.zip › Supplemental Materials/original blots/Fig4R/GAPDH.jpg]

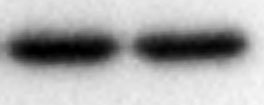

Supplement: Supplementary file 1 — Additional file 1. [file 12944_2023_1891_MOESM1_ESM.zip › Supplemental Materials/original blots/Fig7/Fig7A GAPDH.jpg]

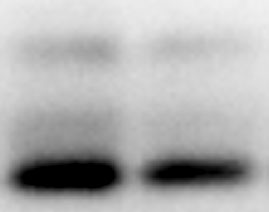

Supplement: Supplementary file 1 — Additional file 1. [file 12944_2023_1891_MOESM1_ESM.zip › Supplemental Materials/original blots/Fig7/Fig7A LC3.jpg]

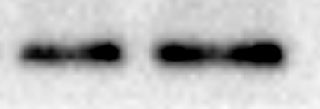

Supplement: Supplementary file 1 — Additional file 1. [file 12944_2023_1891_MOESM1_ESM.zip › Supplemental Materials/original blots/Fig7/Fig7A P62-1.jpg]

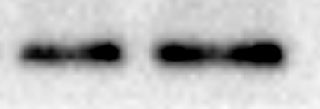

Supplement: Supplementary file 1 — Additional file 1. [file 12944_2023_1891_MOESM1_ESM.zip › Supplemental Materials/original blots/Fig7/Fig7A P62.jpg]

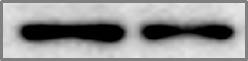

Supplement: Supplementary file 1 — Additional file 1. [file 12944_2023_1891_MOESM1_ESM.zip › Supplemental Materials/original blots/Fig7/Fig7A SNF2H.jpg]

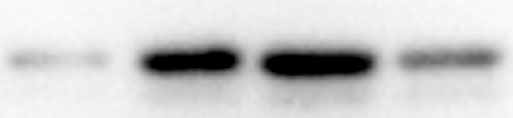

Supplement: Supplementary file 1 — Additional file 1. [file 12944_2023_1891_MOESM1_ESM.zip › Supplemental Materials/original blots/Fig7/Fig7G Adipophilin-1.jpg]

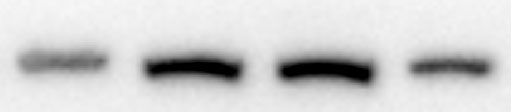

Supplement: Supplementary file 1 — Additional file 1. [file 12944_2023_1891_MOESM1_ESM.zip › Supplemental Materials/original blots/Fig7/Fig7G Adipophilin-2.jpg]

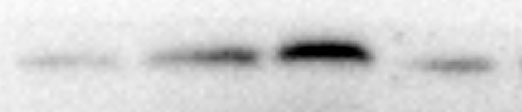

Supplement: Supplementary file 1 — Additional file 1. [file 12944_2023_1891_MOESM1_ESM.zip › Supplemental Materials/original blots/Fig7/Fig7G Adipophilin-3.jpg]

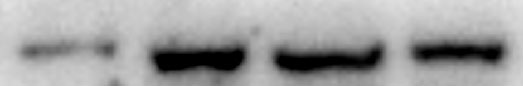

Supplement: Supplementary file 1 — Additional file 1. [file 12944_2023_1891_MOESM1_ESM.zip › Supplemental Materials/original blots/Fig7/Fig7G CATENIN4-1341.5-.jpg]

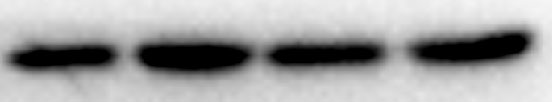

Supplement: Supplementary file 1 — Additional file 1. [file 12944_2023_1891_MOESM1_ESM.zip › Supplemental Materials/original blots/Fig7/Fig7G GAPDH.jpg]

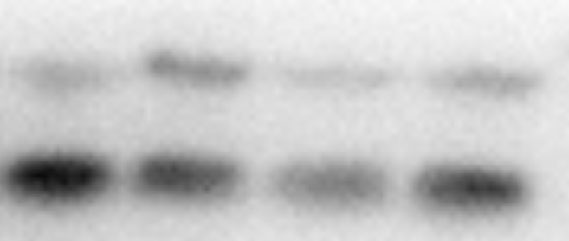

Supplement: Supplementary file 1 — Additional file 1. [file 12944_2023_1891_MOESM1_ESM.zip › Supplemental Materials/original blots/Fig7/Fig7G LC3.jpg]

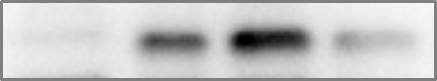

Supplement: Supplementary file 1 — Additional file 1. [file 12944_2023_1891_MOESM1_ESM.zip › Supplemental Materials/original blots/Fig7/Fig7G P62-1.jpg]

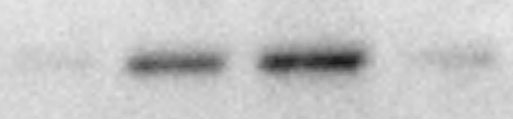

Supplement: Supplementary file 1 — Additional file 1. [file 12944_2023_1891_MOESM1_ESM.zip › Supplemental Materials/original blots/Fig7/Fig7G P62-2.jpg]

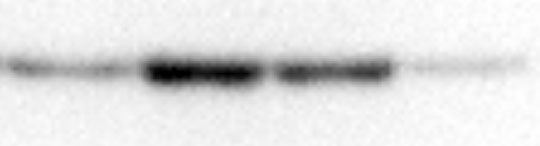

Supplement: Supplementary file 1 — Additional file 1. [file 12944_2023_1891_MOESM1_ESM.zip › Supplemental Materials/original blots/Fig7/Fig7G P62-3.jpg]

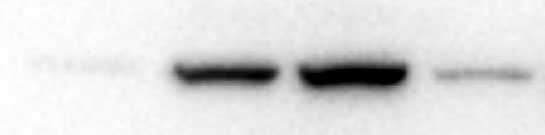

Supplement: Supplementary file 1 — Additional file 1. [file 12944_2023_1891_MOESM1_ESM.zip › Supplemental Materials/original blots/Fig7/Fig7G PLIN2-1.jpg]

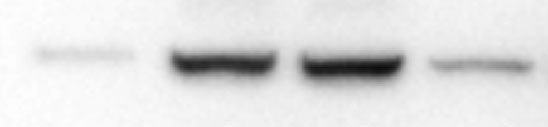

Supplement: Supplementary file 1 — Additional file 1. [file 12944_2023_1891_MOESM1_ESM.zip › Supplemental Materials/original blots/Fig7/Fig7G PLIN2-2.jpg]

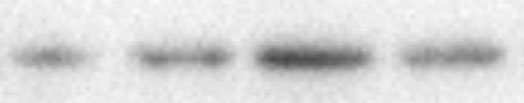

Supplement: Supplementary file 1 — Additional file 1. [file 12944_2023_1891_MOESM1_ESM.zip › Supplemental Materials/original blots/Fig7/Fig7G PLIN2-3.jpg]

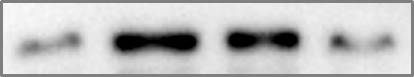

Supplement: Supplementary file 1 — Additional file 1. [file 12944_2023_1891_MOESM1_ESM.zip › Supplemental Materials/original blots/Fig7/Fig7G SNF2H-1.jpg]

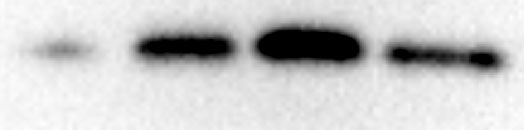

Supplement: Supplementary file 1 — Additional file 1. [file 12944_2023_1891_MOESM1_ESM.zip › Supplemental Materials/original blots/Fig7/Fig7G Wnt1-1.jpg]

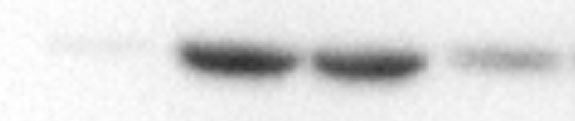

Supplement: Supplementary file 1 — Additional file 1. [file 12944_2023_1891_MOESM1_ESM.zip › Supplemental Materials/original blots/Fig7/Fig7G Wnt1-2.jpg]

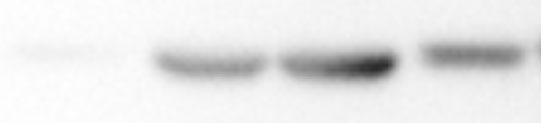

Supplement: Supplementary file 1 — Additional file 1. [file 12944_2023_1891_MOESM1_ESM.zip › Supplemental Materials/original blots/Fig7/Fig7G Wnt1-3.jpg]

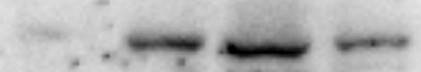

Supplement: Supplementary file 1 — Additional file 1. [file 12944_2023_1891_MOESM1_ESM.zip › Supplemental Materials/original blots/Fig7/Fig7G β-catenin-1.jpg]

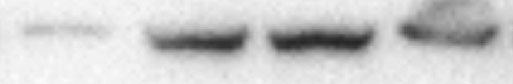

Supplement: Supplementary file 1 — Additional file 1. [file 12944_2023_1891_MOESM1_ESM.zip › Supplemental Materials/original blots/Fig7/Fig7G β-catenin-2.jpg]

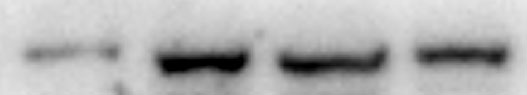

Supplement: Supplementary file 1 — Additional file 1. [file 12944_2023_1891_MOESM1_ESM.zip › Supplemental Materials/original blots/Fig7/Fig7G β-catenin-3.jpg]

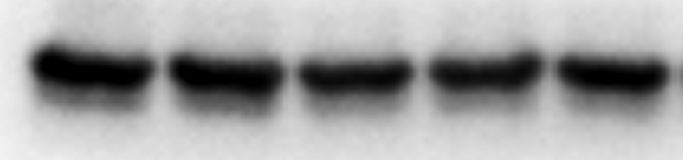

Supplement: Supplementary file 1 — Additional file 1. [file 12944_2023_1891_MOESM1_ESM.zip › Supplemental Materials/original blots/Supplementary Fig1/GAPDH.jpg]

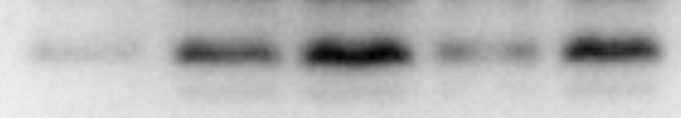

Supplement: Supplementary file 1 — Additional file 1. [file 12944_2023_1891_MOESM1_ESM.zip › Supplemental Materials/original blots/Supplementary Fig1/Supplementary Fig1 Adipophilin-1.jpg]

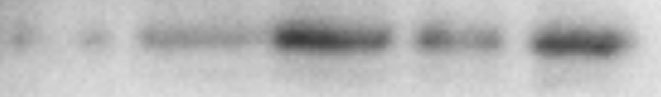

Supplement: Supplementary file 1 — Additional file 1. [file 12944_2023_1891_MOESM1_ESM.zip › Supplemental Materials/original blots/Supplementary Fig1/Supplementary Fig1 Adipophilin-2.jpg]

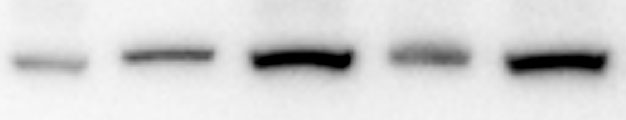

Supplement: Supplementary file 1 — Additional file 1. [file 12944_2023_1891_MOESM1_ESM.zip › Supplemental Materials/original blots/Supplementary Fig1/Supplementary Fig1 Adipophilin-3.jpg]

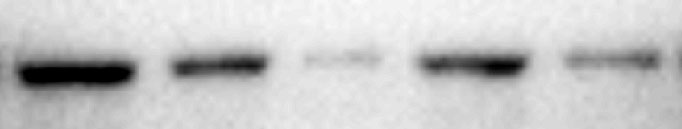

Supplement: Supplementary file 1 — Additional file 1. [file 12944_2023_1891_MOESM1_ESM.zip › Supplemental Materials/original blots/Supplementary Fig1/Supplementary Fig1 Beclin1-1.jpg]

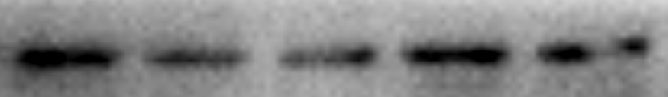

Supplement: Supplementary file 1 — Additional file 1. [file 12944_2023_1891_MOESM1_ESM.zip › Supplemental Materials/original blots/Supplementary Fig1/Supplementary Fig1 Beclin1-2.jpg]

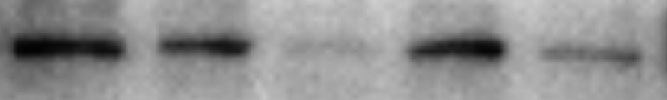

Supplement: Supplementary file 1 — Additional file 1. [file 12944_2023_1891_MOESM1_ESM.zip › Supplemental Materials/original blots/Supplementary Fig1/Supplementary Fig1 LAMP1.jpg]

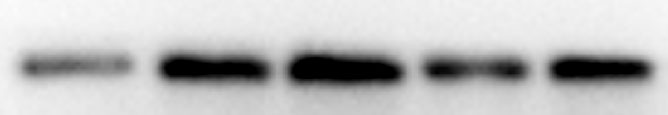

Supplement: Supplementary file 1 — Additional file 1. [file 12944_2023_1891_MOESM1_ESM.zip › Supplemental Materials/original blots/Supplementary Fig1/Supplementary Fig1 P62-1.jpg]

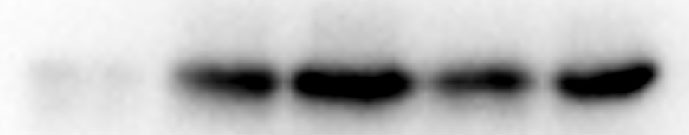

Supplement: Supplementary file 1 — Additional file 1. [file 12944_2023_1891_MOESM1_ESM.zip › Supplemental Materials/original blots/Supplementary Fig1/Supplementary Fig1 P62-2.jpg]

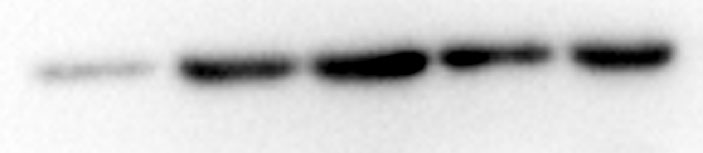

Supplement: Supplementary file 1 — Additional file 1. [file 12944_2023_1891_MOESM1_ESM.zip › Supplemental Materials/original blots/Supplementary Fig1/Supplementary Fig1 P62-3.jpg]

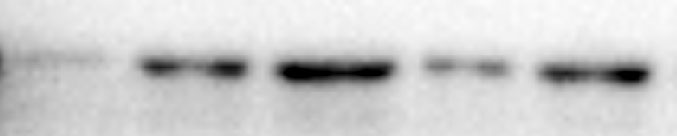

Supplement: Supplementary file 1 — Additional file 1. [file 12944_2023_1891_MOESM1_ESM.zip › Supplemental Materials/original blots/Supplementary Fig1/Supplementary Fig1 PLIN2-1.jpg]

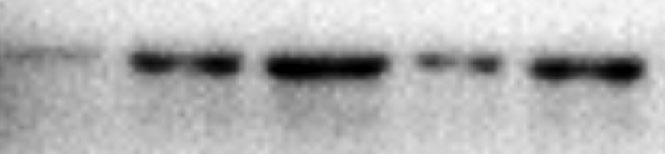

Supplement: Supplementary file 1 — Additional file 1. [file 12944_2023_1891_MOESM1_ESM.zip › Supplemental Materials/original blots/Supplementary Fig1/Supplementary Fig1 PLIN2-2.jpg]

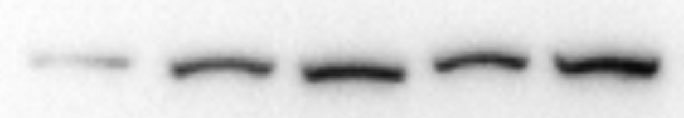

Supplement: Supplementary file 1 — Additional file 1. [file 12944_2023_1891_MOESM1_ESM.zip › Supplemental Materials/original blots/Supplementary Fig1/Supplementary Fig1 PLIN2-3.jpg]

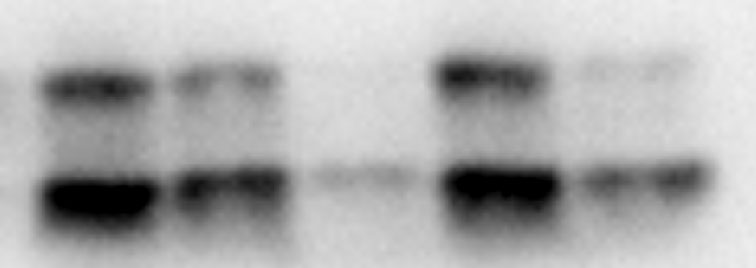

Supplement: Supplementary file 1 — Additional file 1. [file 12944_2023_1891_MOESM1_ESM.zip › Supplemental Materials/original blots/Supplementary Fig1/Supplementary Fig1A LC3-1.jpg]

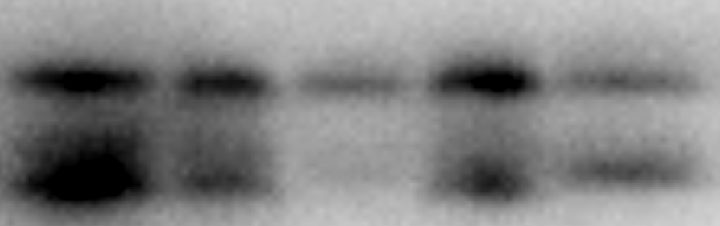

Supplement: Supplementary file 1 — Additional file 1. [file 12944_2023_1891_MOESM1_ESM.zip › Supplemental Materials/original blots/Supplementary Fig1/Supplementary Fig1A LC3-2.jpg]

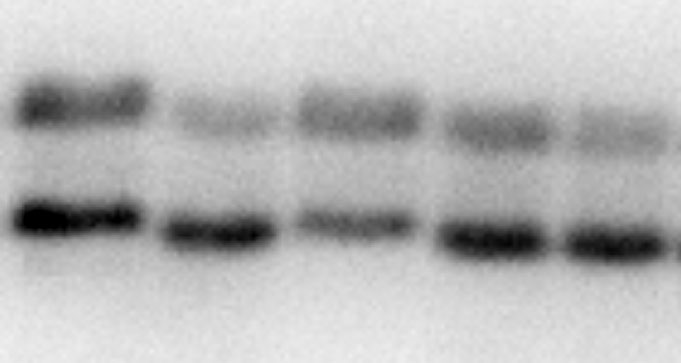

Supplement: Supplementary file 1 — Additional file 1. [file 12944_2023_1891_MOESM1_ESM.zip › Supplemental Materials/original blots/Supplementary Fig1/Supplementary Fig1A LC3-3.jpg]

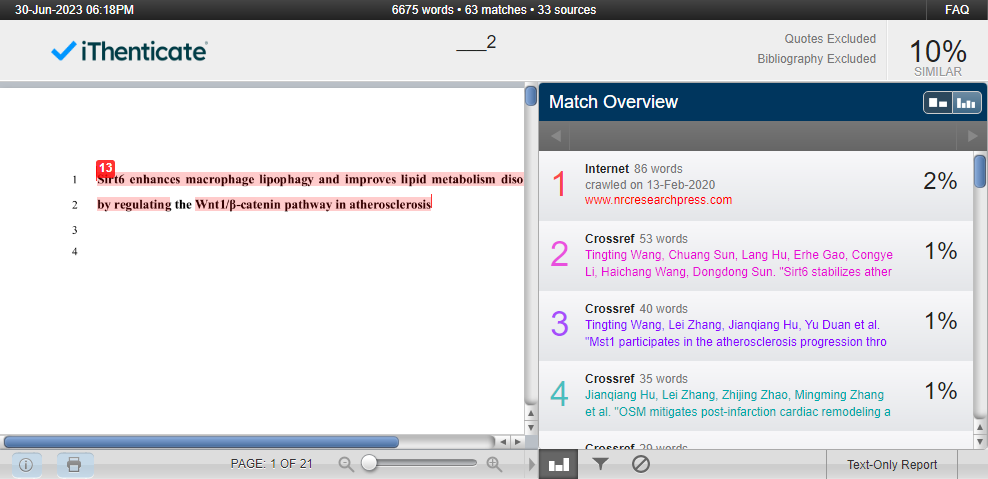

Supplement: Supplementary file 1 — Additional file 1. [file 12944_2023_1891_MOESM1_ESM.zip › Supplemental Materials/similarity index.png]

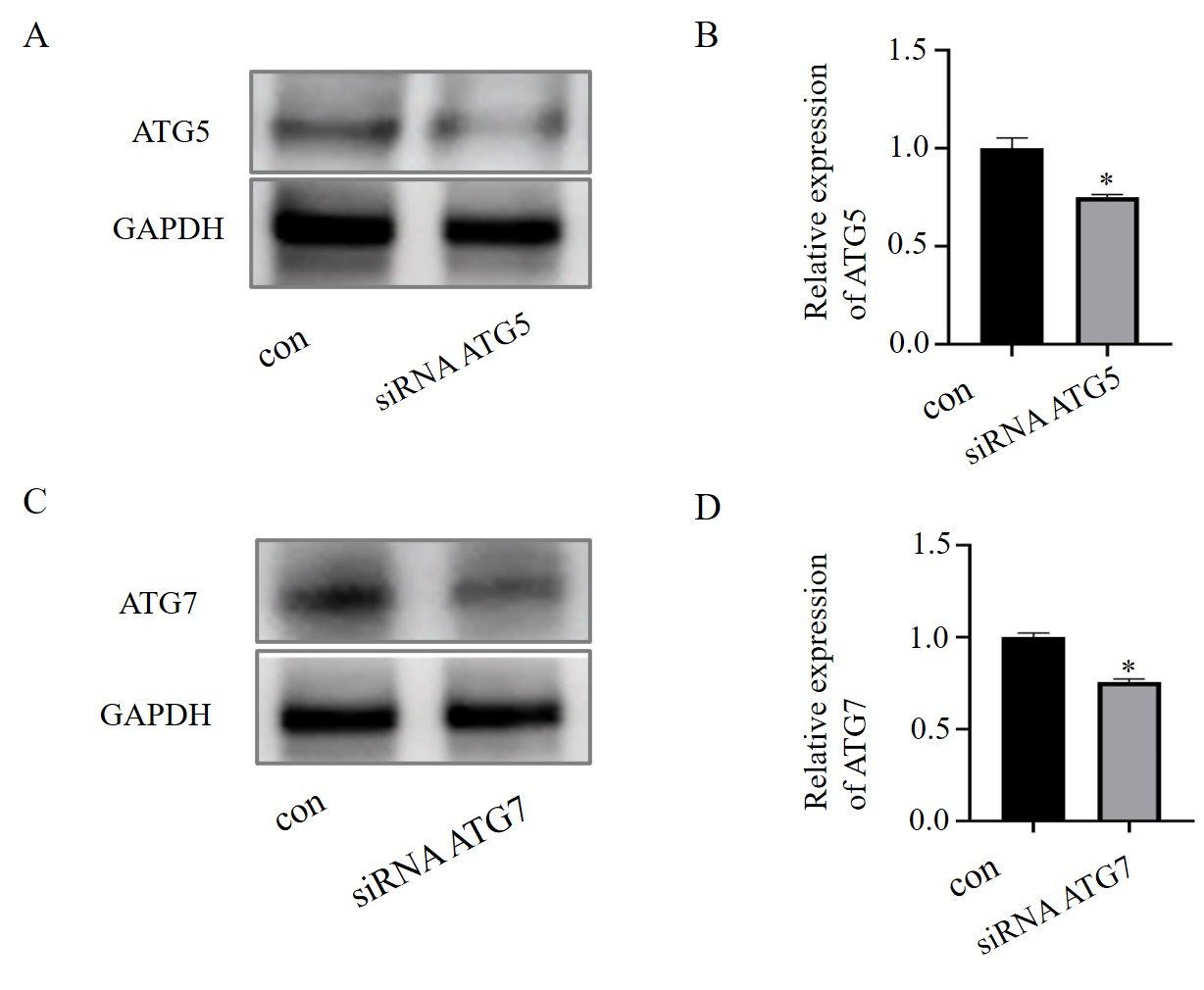

Supplement: Supplementary file 2 — Additional file 2: Supplementary Figure 1. siRNA ATG5 and siRNA ATG7 worked by western blot. (A, C) Expression of ATG5 and ATG7 treated with siRNA ATG5 and siRNA ATG7 were evaluated by western blot; (B, D) Quantitative analysis of ATG5 and ATG7 expression by western blot. Data are expressed as mean ± SE (Student’s t-test showed that there was statistical significance between Con group and siRNA ATG5 with siRNA ATG7 group. * P < 0.01 vs the control group [n = 6]). [file 12944_2023_1891_MOESM2_ESM.jpg]

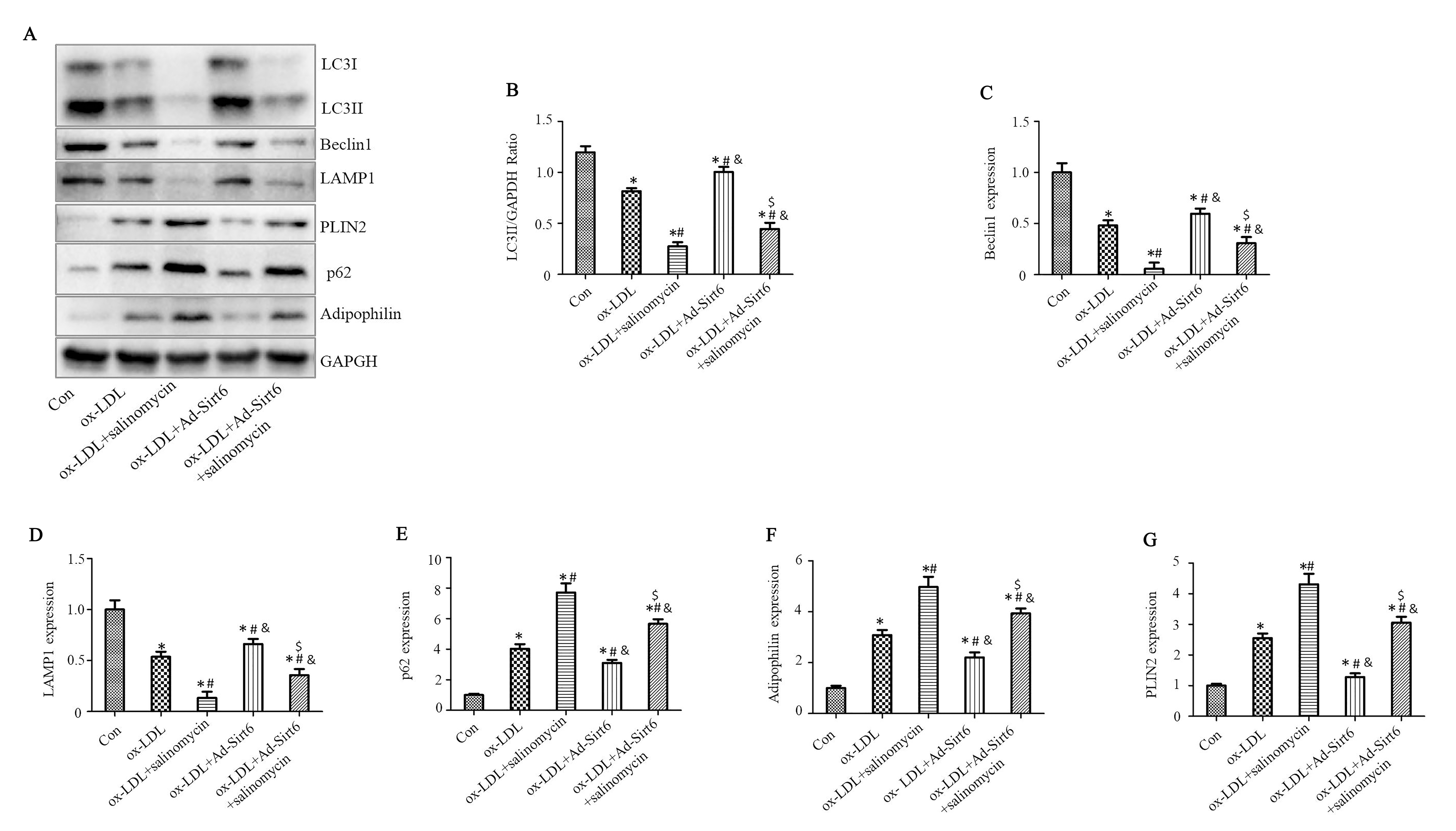

Supplement: Supplementary file 3 — Additional file 3: Supplementary Figure 2. Sirt6 regulates lipophagy by inhibiting Wnt1. (A–G) Analysis of LC3, Beclin1, LAMP1, adipophilin, PLIN2, and P62 by immunoblotting and quantitative analysis (n = 6). Data are expressed as mean ± SE (One way ANOVA showed that there was statistical significance among groups. * P < 0.05 vs the control group; #P < 0.05 vs the ox-LDL group; &P < 0.05 vs the Ac-LDL+salinomycin group; $P < 0.05 vs the ox-LDL+Ad-Sirt6 group [n = 6]). [file 12944_2023_1891_MOESM3_ESM.jpg]

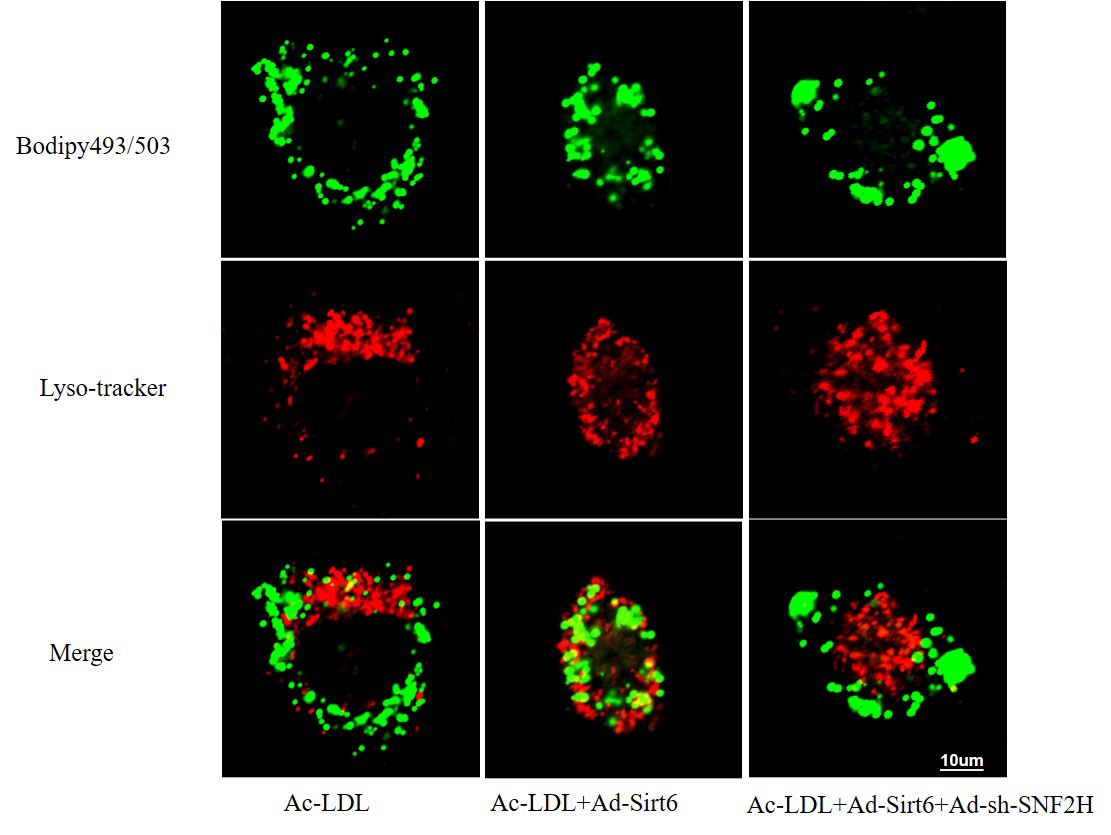

Supplement: Supplementary file 4 — Additional file 4: Supplementary Figure 3. The effect of Sirt6 on lipid droplets degradation was SNF2H dependent. (A) Co-localization of lipid droplets and lysosomes analyzed by LysoTracker Red and BODIPY 493/503 staining were detected by confocal microscopy in the presence or absence of SNF2H administration (n=6 separate experiments). [file 12944_2023_1891_MOESM4_ESM.jpg]
